# Ribosome engineering reveals the importance of 5S rRNA autonomy for ribosome assembly

**DOI:** 10.1038/s41467-020-16694-8

**Published:** 2020-06-09

**Authors:** Shijie Huang, Nikolay A. Aleksashin, Anna B. Loveland, Dorota Klepacki, Kaspar Reier, Amira Kefi, Teresa Szal, Jaanus Remme, Luc Jaeger, Nora Vázquez-Laslop, Andrei A. Korostelev, Alexander S. Mankin

**Affiliations:** 1 Center for Biomolecular Sciences, University of Illinois at Chicago, Chicago, IL 60607 USA; 2 Department of Pharmaceutical Sciences, University of Illinois at Chicago, Chicago, IL 60607 USA; 3 RNA Therapeutics Institute, Department of Biochemistry and Molecular Pharmacology, University of Massachusetts Medical School, 368 Plantation St., Worcester, MA 01605 USA; 4 Institute of Molecular and Cellular Biology, University of Tartu, Riia 23, 51010 Tartu, Estonia; 5 Chemistry and Biochemistry Department, University of California, Santa Barbara, CA 93106-9510 USA; 6 Present Address: Department of Chemistry, University of Pennsylvania, Philadelphia, PA USA; 7 Present Address: Department of Molecular and Cell Biology, University of California, Berkeley, CA USA

**Keywords:** RNA, Cryoelectron microscopy, Ribosome

## Abstract

5S rRNA is an indispensable component of cytoplasmic ribosomes in all species. The functions of 5S rRNA and the reasons for its evolutionary preservation as an independent molecule remain unclear. Here we used ribosome engineering to investigate whether 5S rRNA autonomy is critical for ribosome function and cell survival. By linking circularly permutated 5S rRNA with 23S rRNA we generated a bacterial strain devoid of free 5S rRNA. Viability of the engineered cells demonstrates that autonomous 5S rRNA is dispensable for cell growth under standard conditions and is unlikely to have essential functions outside the ribosome. The fully assembled ribosomes carrying 23S-5S rRNA are highly active in translation. However, the engineered cells accumulate aberrant 50S subunits unable to form stable 70S ribosomes. Cryo-EM analysis revealed a malformed peptidyl transferase center in the misassembled 50S subunits. Our results argue that the autonomy of 5S rRNA is preserved due to its role in ribosome biogenesis.

## Abstract (web summary)

Ribosomes of all organisms have retained 5S rRNA as an autonomous rRNA species. Here the authors engineer a bacterial strain with ribosomes that do not have free 5S rRNA, and carry structural analyses that suggest the evolutionary preservation of 5S rRNA as an independent molecule is based on its role in the dynamic process of ribosome biogenesis.

## Introduction

**Fig. 1:**
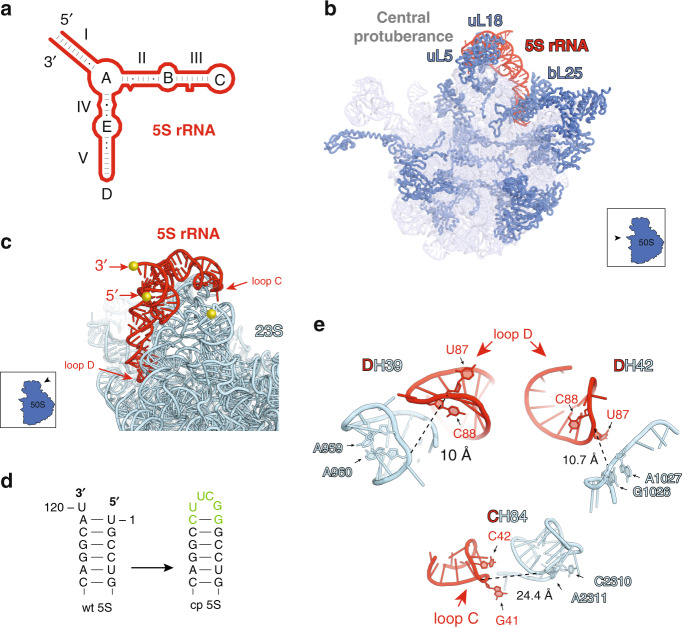
5S rRNA in the ribosome. **a** Secondary structure of the 5S rRNA; RNA stems (I-V) and loops (A–E) are indicated. **b** The view of the *E. coli* 50S ribosomal subunit from the interface side (PDB 4YBB^7^). 5S rRNA is shown in red, 23S rRNA is light blue and r-proteins in dark blue. **c** Interactions between wt 5S rRNA and 23S rRNA (as seen from the large subunit solvent side). The native ends of the 5S rRNA and the nearest accessible 23S rRNA site are marked by golden spheres. Loops C and D, which are in a close proximity to 23S rRNA, are indicated. Proteins are removed for clarity. **d** Engineering cp5S rRNA by linking its native ends with a tetra-loop. The altered/added residues are in green. **e** The cp5S rRNA integration sites. The shortest distances between the nucleotide phosphates of the 5S rRNA and 23S rRNA are indicated by dotted lines.

Cytoplasmic ribosomes of all species contain highly-conserved 5S rRNA. The reason for the evolutionary preservation of this smallest rRNA as an independent molecule or its general role in translation remain enigmatic. The secondary structure of the 5S rRNA is conserved across all species^1^ and consists of five helices (I-V) and five loops (A-E) (Fig. 1a). In the bacterial (*Escherichia coli*) ribosome, 5S rRNA interacts with three r-proteins uL5, uL18, and bL25 (Fig. 1b)^2–4^. Together with these and few other r-proteins as well as elements of domains II and V of 23S rRNA, 5S rRNA forms a distinct feature of the large ribosomal subunit, known as the central protuberance (Fig. 1b). In the fully assembled subunit, 5S rRNA is proximal to the key functional elements of the ribosome, including the GTPase-associated center (GAC) and peptidyl transferase center (PTC)^3,5–7^.

Classic in vitro reconstitution experiments pointed to a possible role of 5S rRNA in the PTC function because large (50S) subunits reconstituted in the absence of 5S rRNA were deprived of peptidyl transferase activity^2,8–10^. However, subsequent experiments showed that the presence of macrolide antibiotics during in vitro subunit assembly could partially compensates for the lack of 5S rRNA and restore peptidyl transferase activity arguing against direct involvement of 5S rRNA in the functions of the PTC^11^. It has also been suggested that 5S rRNA participates in the communication between PTC and other functional centers of the ribosome and in maintaining the fidelity of translation^2,12–14^. More recent cryogenic electron microscopy (cryo-EM) reconstructions showed that 5S rRNA may undergo an extensive ~180° rotation during 50S assembly, possibly critical for the maturation of the central protuberance, PTC and GAC^15,16^.

Preservation of the autonomy of 5S rRNA might also point to its putative extraribosomal functions. In more than 20% of the sequenced bacterial genomes, the 5S gene dosage exceeds that of the 16S and 23S rRNA genes, even though all rRNA molecules are present in equimolar amounts in the ribosome^17^. For example, the genome of *Syntrophomonas wolfei* carries three copies of 16S and 23S rRNA genes but features thirteen 5S rRNA gene copies^18^. In *E. coli*, six out of the seven rRNA operons are composed of one copy each of 16S, 23S, and 5S rRNA genes, while the *rrnD* operon carries an additional copy of the 5S rRNA gene. The surplus of 5S relative to 16S/23S rRNA genes in some bacterial genomes could indicate important activities of 5S rRNA outside of the ribosome, resembling well-documented moonlighting functions of some r-proteins^19,20^. In agreement with this possibility, deletion of 5S rRNA genes in *E. coli* results in a more pronounced loss of fitness than the elimination of the same number of 16S and 23S rRNA genes^21^. The possibility that 5S rRNA could have extraribosomal functions in eukaryotes is suggested by a proposed regulatory role of 5S rRNA in tumor suppression^22–26^ and by the transient export of 5S rRNA into the cytoplasm of eukaryotic cells prior to its eventual return to the nucleus for incorporation into the large ribosomal subunit^27,28^.

To test whether maintaining 5S rRNA as an autonomous molecule is essential for protein synthesis, ribosome assembly, or for extraribosomal functions of this rRNA, we engineered an *E. coli* strain devoid of free 5S rRNA. This was achieved by integrating circularly permutated 5S (cp5S) rRNA into 23S rRNA and deleting all wild type (wt) 5S rRNA genes. The viability of the cells lacking free 5S rRNA rules out the existence of essential extraribosomal functions of this molecule in bacteria. Our functional and structural analyses of the engineered ribosomes reveal that the autonomous nature of 5S rRNA is not required for the ribosome ability to synthesize proteins and is not related to any essential extraribosomal functions. Instead, the autonomy of 5S rRNA is likely evolutionarily preserved due to its role in ribosome assembly.

## Results

### Engineering hybrid 23S-cp5S rRNA

To test whether the autonomy of 5S rRNA is a prerequisite for ribosome assembly, ribosome function, and/or cell viability, we integrated the 5S rRNA sequence into 23S rRNA to create a hybrid 23S-cp5S rRNA molecule. In the structure of the wt ribosome, the native ends of the 5S rRNA are distant from the nearest 23S rRNA site (Fig. 1c) and, hence, the shortest RNA linker connecting them to the 23S rRNA would have to be at least 50 Å long. Such a long RNA tether would likely be susceptible to nuclease cleavage and thus, inappropriate for our goals. To avoid this problem, we took advantage of the spatial proximity of the 5′ and 3′ termini of 5S rRNA (Fig. 1a, c), which makes this molecule suitable for circular permutations. By connecting the native ends of 5S rRNA (Fig. 1d) and ‘opening’ the structure at a new location we could potentially integrate the resulting cp5S rRNA sequence into 23S rRNA proximal sites using short tethers.

In order to preserve the evolutionary conserved structure of the cp5S rRNA, we introduced new ends at the apex loops C or D located in the proximity to the 23S rRNA (Fig. 1a, c). Loop C of 5S rRNA is within a short distance from the loop of H84 of the 23S rRNA, whereas loop D closely approaches the H39 loop and the base of H42 (Fig. 1e). Integration of cp5S rRNA into any of these 23S rRNA sites would require very short (0–3 nt long) RNA tethers.

**Fig. 2:**
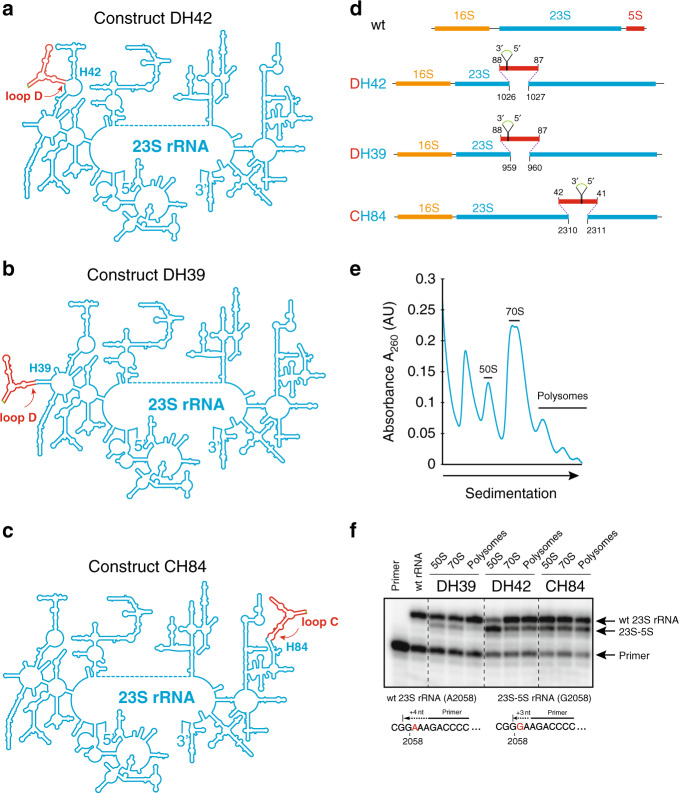
Engineering 23S-cp5S rRNA. **a**–**c** Secondary structures of the three engineered 23S-cp5S rRNA constructs DH42 (**a**), DH39 (**b**) and CH84 (**c**). 23S rRNA is in blue and integrated cp5S rRNA is in red; the connector linking 5′ and 3′ end of wt 5S rRNA is in green. **d** Schematic representation of the structures of wt and engineered 23S-cp5S rRNA operons. In cp5S, native 5′ and 3′ 5S rRNA ends are linked by a 4-nt connector (green). The resulting cp5S rRNA is “opened” in loop D (constructs DH42 and DH39) or loop C (construct CH84) and inserted via short tethers (dotted lines) connecting the indicated positions of the 23S rRNA and 5S rRNA. **e** Sucrose gradient separation of the ribosomal material from *E. coli* POP2136 cells expressing a mixed population of wt and 23S-cp5S ribosomes (construct DH42). The fractions used for preparation of the RNA from the large subunits (50S), ribosomes (70S), and polysomes are indicated. **f** Primer extension analysis of the 50S, 70S and polysomal rRNA prepared from randomly picked clones transformed with pDH42, pDH39, or pCH84 plasmids. In the presence of ddCTP, the primer is extended by 4 nt on the wt (A2058) rRNA template or by 3 nt on the 23S-cp5S rRNA template that contains the A2058G mutation. The first two lanes were loaded with the control samples: the radiolabeled primer (first lane) and primer extension products obtained with wt 23S rRNA. Note the presence of the mutant rRNA-specific band in the polysome fractions of the DH42 and CH84 constructs. The uncropped gel can be found in the Source data file. The results shown in **e** and **f** are typical representatives of 2 independent experiments.

We prepared three libraries of DNA constructs, DH39, DH42, and CH84 (Fig. 2a–d) corresponding to the three designs. The constructs were engineered using the plasmid pAM552, which contains the intact *E. coli rrnB* operon (Supplementary Fig. 1a)^29^. First, to facilitate the selection and identification of the functional variants, the A2058-to-G mutation was introduced into the 23S rRNA-coding sequence, rendering mutant ribosomes resistant to erythromycin (Ery)^30^. Then, the wt 5S rRNA gene was deleted from the plasmid-borne rRNA operon. Finally, cp5S rRNA sequences were introduced into one of the three aforementioned sites in the 23S rRNA gene (Fig. 2a–d). Because it was difficult to predict the optimal length and sequence of the cp5S-23S rRNA linkers, random sequence 5′ and 3′ tethers, ranging in lengths from 0 to 3 nucleotides, were used to insert cp5S rRNA into 23S rRNA hypothetically yielding 7225 tether combinations for each of the three designs.

### The 23S-cp5S rRNA can form functionally active ribosomes

We first tested whether hybrid 23S-cp5S rRNA ribosomes can be expressed in wt *E. coli* cells and form functional ribosomes. The plasmid libraries, corresponding to the three 23S-cp5S chimeric constructs DH39, DH42, and CH84 (Fig. 2a–c) were transformed into the *E. coli* strain POP2136^31^. Primer extension analysis performed with several randomly picked pDH39, pDH42, and pCH84 transformants revealed the presence of the hybrid 23S-cp5S rRNA in the 50S and 70S fractions. Importantly, in the DH42 and CH84 clones, the hybrid rRNA was also prominent in polysomes (Fig. 2e, f) demonstrating that the hybrid 23S-cp5S rRNA can form translationally active 70S ribosomes. These results encouraged us to ask whether the engineered ribosomes are sufficiently active to support cell growth even in the absence of wt ribosomes and free 5S rRNA.

### Generating a bacterial strain devoid of free 5S rRNA

**Fig. 3:**
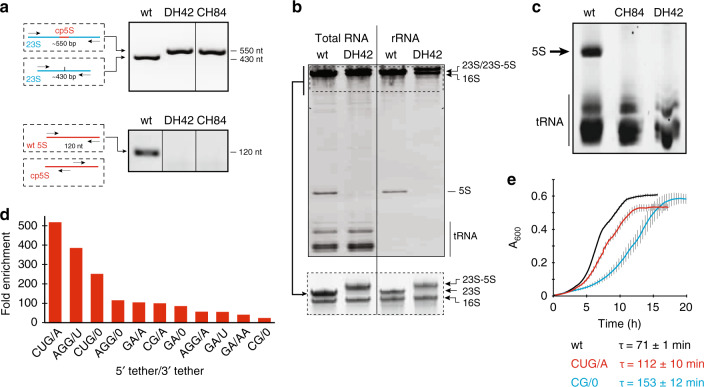
The 23S-cp5S ribosomes support cell growth in the absence of free 5S rRNA. **a** PCR analysis of rDNA in the *E. coli* SQA18 cells expressing wt 23S rRNA or chimeric 23S-cp5S rRNAs. Top: PCR with 23S rRNA-specific primers (black) flanking the site of cp5S rRNA (red) insertion into 23S rRNA (blue). Bottom: PCR with 5S rRNA-specific primers (black) annealing to the ends of wt 5S rRNA gene (red) generate no amplified product when DNA from the DH42 or CH84 clones is used as a template. **b** Polyacrylamide gel electrophoresis of total RNA or rRNA isolated from wt or SQA18/DH42 cells. Bottom: same samples as above but running electrophoresis for an extended time to improve separation of the large rRNA species. **c** Gel electrophoresis analysis of small RNA extracted from wt, SQA18/CH84, or SQA18/DH42 cells cured of the pCSacB plasmid. The gel was purposefully overloaded to confirm the lack of free 5S rRNA in the CH84 and DH42 clones. **d** Identification of the best linker combinations for the 23S-cp5S DH42 construct. The bar graph shows the fold enrichment of the indicated linker pairs in the selected library relative to the unselected one. **e** Growth of the *E. coli* SQA18 strain expressing wt ribosomes (black line) or ribosomes containing 23S-cp5S variants DH42(CUG/A) (referred as DH42*) (red line) and DH42(CG/0) (blue line). Each curve is an average of three replicates, with error bars indicating s.d. Calculated doubling time (τ) of the strains is indicated. The representative gels of at least two independent experiments are shown in **a**–**c**.

Subsequent experiments were carried out in the engineered *E. coli* strain SQA18 that lacks chromosomal rRNA alleles, including all 5S rRNA genes, and expresses wt rRNA from the pCSacB plasmid^32^ (for details see Methods, Supplementary Table 1 and Supplementary Fig. 1c). After transformation of the SQA18 cells with the plasmid libraries DH39, DH42 and CH84 the pCSacB plasmid was eliminated by counterselection. Viable clones lacking pCSacB were obtained with the DH42 and CH84 libraries, indicating that mutant ribosomes with cp5S rRNA integrated at either H42 or H84 of 23S rRNA can support cell growth in the absence of wt ribosomes. PCR analysis of randomly picked DH42 and CH84 clones verified the lack of wt 5S rRNA gene (Fig. 3a) and examination of total cellular RNA, as well as rRNA extracted from the isolated ribosomes, confirmed the absence of free 5S rRNA (Fig. 3b, c). Gel electrophoresis also revealed the mobility shift of the large rRNA reflecting the insertion of the 120 nt-long cp5S rRNA sequence into 23S rRNA (Fig. 3b). To the best of our knowledge, the engineered *E. coli* strains represent the first examples of free-living cells lacking autonomous 5S rRNA.

The heterogeneity of the sizes of the DH42 and CH84 library colonies suggested that the nature of the 23S-cp5S rRNA tethers affects the growth characteristics of the strains and possibly the functionality or assembly of the engineered ribosomes. Therefore, we proceeded to identify the optimal tethers in the DH42 library, which had demonstrated more efficient wt pCSacB plasmid loss in the pilot experiments. By deep-sequencing the SQA18 DH42 transformants before and after counterselection of the resident pCSacB plasmid we identified several tether variants that were significantly enriched in the cells that readily lost the plasmid encoding wt rRNA. The three most prevalent tether combinations showed more than a 200-fold enrichment (Fig. 3d and Source data file). They all contained a 3-nt linker at the 5′ end of the cp5S insert. By contrast, 0-length or a 1 nt-long linker were favored at the 3′ end (Fig. 3d). Consistent with the expectations that the more prevalent 23S-cp5S linkers would increase cell fitness, the clone carrying the winning tether pair 5′ CUG-cp5S-A 3′ (518-fold enrichment) grew notably faster than cells carrying the less optimal 5′ CG-cp5S-0 3′ tethers (24-fold enrichment) (Fig. 3d, e). Although the doubling time of even the winning CUG/A variant (112 ± 10 min) was ~1.6 times longer than the generation time of the SQA18 cells expressing wt ribosomes, the robust growth of cells lacking free 5S rRNA argued that this molecule is likely not involved in any essential extraribosomal function in the bacterial cell. For all the subsequent experiments, we chose the fastest-growing DH42(CUG/A) clone. Throughout the rest of the paper we will refer to this clone as DH42*.

### 23S-cp5S ribosomes retain high translation activity

We were wondering whether the slower growth of the DH42* cells lacking free 5S rRNA reflects suboptimal performance of the ribosomes with the hybrid 23S-cp5S rRNA in translating the *E. coli* proteome. Therefore, we isolated mutant 70S ribosomes and tested their activity in a cell-free translation system.

**Fig. 4:**
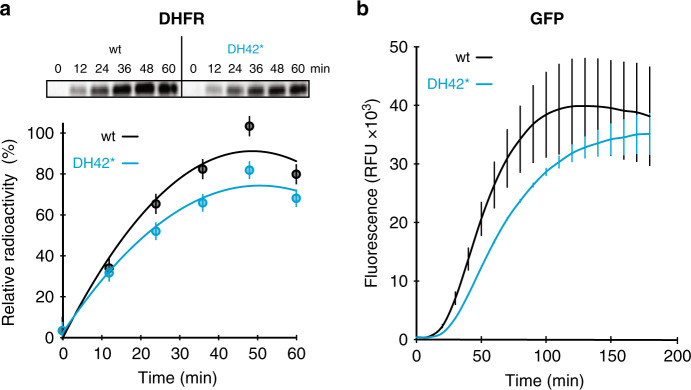
Functional ribosomes with the hybrid 23S-cp5S rRNA are highly active in translation. **a** In vitro translation of dihydrofolate reductase (DHFR) protein by wt ribosomes or23S-cp5S rRNA DH42* ribosomes. Top: SDS–gel analysis of [^35^S]-labeled DHFR protein synthesized in the cell-free translation system. The intensities of the full-size protein bands were plotted in the graph shown below the gel (mutant—blue curve, wt—black curve). **b** Time course of in vitro expression of sfGFP protein by wt or mutant ribosomes as evaluated by fluorescence (mutant—blue curve, wt—black curve). Graphs shown in **a** and **b** represent the average of three independent experiments with error bars indicating s.d. The uncropped gels and raw data can be found in the Source data file.

Two unrelated reporter proteins (dihydrofolate reductase and green fluorescent protein) were synthesized by the mutant ribosomes at a rate approaching that of the wt ribosomes (~80%) (Fig. 4), demonstrating high activity of the ribosomes with the single 23S-cp5S rRNA. Furthermore, the rate of translation by the engineered ribosomes in these assays is likely underestimated due to the contamination of our 70S preparations with aberrant unassociated 50S subunits and non-functional 70S ribosomes (see below).

These results showed that the functionality of the bacterial ribosome does not rely on the autonomous nature of 5S rRNA.

### Aberrant subunits accumulate in the absence of free 5S rRNA

**Fig. 5:**
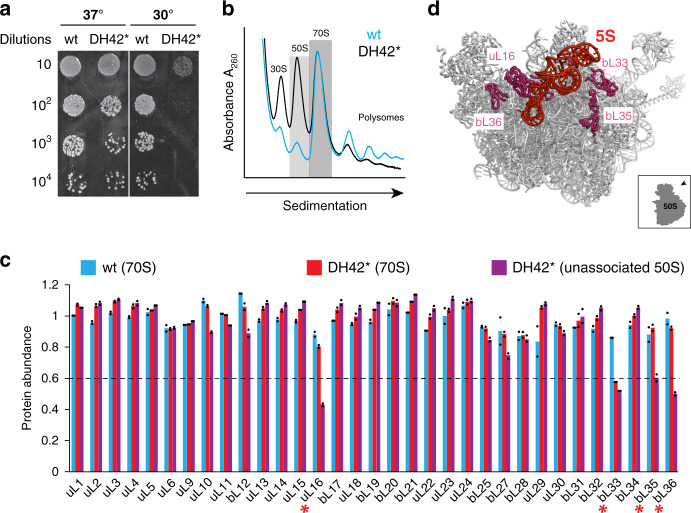
The lack of free 5S rRNA results in assembly defects. **a** Cold sensitivity of cells expressing 23S-cp5S ribosomes. Exponentially-growing cultures of wt or DH42* cells were serially diluted and spotted onto LB/agar plates supplemented with Amp. Plates were incubated at 37 °C for 24 h or at 30 °C for 48 h. **b** Sucrose gradient analysis of ribosomal fractions isolated from wt or 23S-cp5S DH42* cells. Fractions shaded with dark (70S) and light (unassociated 50S subunits) gray boxes were collected for subsequent analysis. **c** Protein composition of the large subunits in wt (blue bars) or mutant (red bars) 70S ribosomes and of the unassociated mutant 50S subunits (purple bars). Red asterisks indicate the r-proteins that are significantly underrepresented (<60% of the remaining amount) in the unassociated 50S subunits. The bar graph represents the mean of two biological replicates with individual data points indicated by black dots. Raw data can be found in the Source Data file. **d** Relative location of the 5S rRNA (red) and underrepresented proteins (purple) in the large subunit structure viewed from the solvent side.

Because free 5S rRNA does not seem to be required for ribosome functions in translation, we surmised that the autonomy of the smallest rRNA might benefit ribosome assembly, which would explain its evolutionary preservation as an independent molecule. Ribosome biogenesis defects are often associated with cold sensitivity^33^. Indeed, while the DH42* strain lacking free 5S rRNA readily grew at 37 °C, it failed to form colonies at 30 °C (Fig. 5a). This observation prompted us to investigate in more detail the assembly of 23S-cp5S rRNA into 50S subunits and 70S ribosomes.

Sucrose gradient centrifugation showed that DH42* cells accumulate notably higher amounts of unassociated 50S and 30S ribosomal subunits than the wt strain (Fig. 5b), indicating that a significant fraction of the large subunits with the hybrid 23S-cp5S rRNA are unable in forming stable 70S ribosome. To characterize the composition of the 50S subunits that remain unassociated, we compared their r-protein content with that of the large subunits of the 70S ribosomes isolated from the same cells. Mass-spectrometry analysis showed that the 70S ribosomes that contained 23S-cp5S rRNA retained the full set of large subunit r-proteins in nearly stoichiometric amounts, except for a 35% underrepresentation of protein bL33 (Fig. 5c). By contrast, the unassociated 50S subunits lacked not only bL33 but also a subset of other late-assembly proteins^34^, most notably the essential protein uL16^35^, as well as bL35 and bL36 (Fig. 5c and Supplementary Fig. 2). Because these proteins bind in the vicinity of the 5S rRNA-containing central protuberance (Fig. 5d), their deficiency likely results from atypical assembly of the 50S subunit with 23S-cp5S rRNA. Thus, while some 70S ribosomes can be formed in the absence of the free 5S rRNA, a notable fraction of the 50S subunits with 23S-cp5S rRNA misassemble into particles with an incomplete set of r-proteins.

### The functional mutant ribosomes are structurally unperturbed

**Fig. 6:**
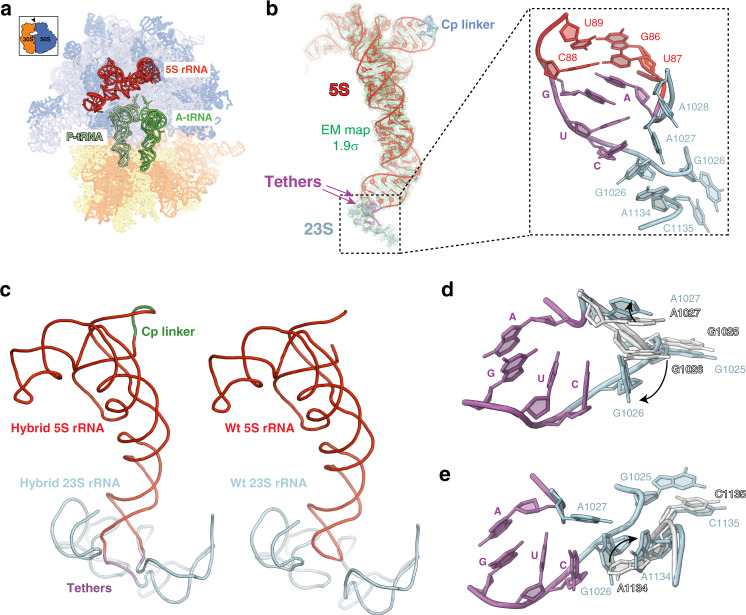
Structure of the active ribosomes with the hybrid 23S-cp5S rRNA. **a** Cryo-EM reconstruction of the 23S-cp5S rRNA 70S ribosome with A and P sites occupied by tRNAs (green). 23S rRNA-linked cp5S rRNA is shown in red. **b** Fitting of tethered cp5S rRNA in the cryo-EM density. cp5S rRNA is red, tethers are purple and 23S rRNA nucleotides are light-blue. Inlet shows the modeled positions of nucleotides involved in forming the cp5S - 23S rRNA junction (see also Supplementary Fig. 4a). **c** The path of the rRNA backbone in wt ribosomes (left panel)^67^ and in ribosomes with hybrid 23S-cp5S rRNA (right panel) deduced by cryo-EM analysis. Colors are as in **b**. **d**, **e** Tethering 23S and cp5S rRNA introduces minimal perturbation at the junction site with only G1026 and A1027 at the site of attachment (**c**) and a more distant A1134 (**d**) undergoing conformational transitions. The orientation of the 23S rRNA residues in the wt ribosomes is shown in gray, 23S rRNA nucleotides of the mutant ribosome are light blue and the RNA residues belonging to the 23S rRNA-cp5S rRNA tethers are purple.

**Fig. 7:**
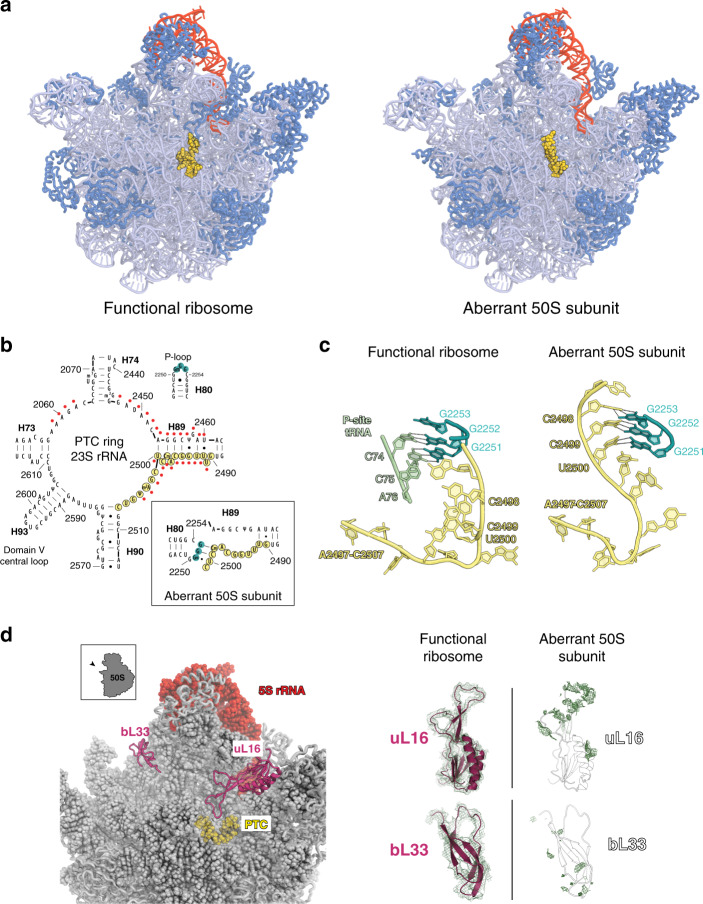
The structure of the aberrant 50S subunits with 23S-cp5S rRNA. **a** Cryo-EM reconstruction of the structure of the 50S subunit in the functional 70S ribosomes (left panel) and unassociated 50S subunit (right panel) containing the 23S-cp5S rRNA. 23S rRNA is light blue, 5S rRNA is red, r-proteins are dark blue. The segments of the PTC rRNA that is remodeled in the aberrant 50S subunit is shown in yellow. **b** The secondary structure diagram of the central ring of domain V of the 23S rRNA that constitutes the heart of the PTC active site. The rRNA residues misplaced in the aberrant 50S subunits are indicated with red dots. The inlet shows the change in the structure at the H89 base: rearrangement of the PTC structure results in the unwinding of the first helical turn of H89 (see also Supplementary Fig. 5b). **c** Interaction of the 23S rRNA P-loop (dark blue) with the CCA end of the P-site bound tRNA (green) in the functional ribosome or 23S rRNA segment 2497–2507 (yellow) in the aberrant 23S-cp5S large ribosomal subunits. rRNA strands are colored as in **b**. In the aberrant 50S subunit, the interaction of the tRNA with P-loop is blocked by the abnormal intramolecular pairing within the 23S rRNA. **d** Proteins uL16 and bL33 are lacking in the aberrant 50S subunits. Left: the location of uL16 and bL33 (purple) in the large subunit of the active 70S ribosome. 5S rRNA is red. The 23S rRNA segment 2490–2507 in the PTC active site (see **b**) is shown in yellow. Right: the cryo-EM density for uL16 and bL33 (green mesh) is largely missing in the aberrant 50S subunits.

We used cryo-EM to examine the structures of the 70S ribosomes and 50S subunits from DH42* cells. While the large subunits collected from the sucrose gradient 50S peak tended to aggregate and were not suitable for cryo-EM grid preparation, the material in the 70S peak contained unassociated 50S subunits in sufficient amounts for structural analysis (Supplementary Fig. 3a). Maximum-likelihood classification of the 489,732-particle dataset resolved 70S and 50S maps at 3.1–3.5 Å average resolution allowing reconstruction of the detailed structures of the ribosomes and large subunits (Figs. 6, 7 and Supplementary Fig. 3).

Nearly one third of the dataset (32%) corresponded to 70S ribosomes carrying one or two tRNA molecules (Supplementary Fig. 3a and Supplementary Table 3). This fraction likely represents active ribosomes. We used 26,717 particles corresponding to the non-rotated ribosomes with the P- and A-site bound tRNAs to solve the structure of the functional ribosome with the 23S-cp5S rRNA (Fig. 6a). These 70S particles are structurally similar to wt ribosomes^7,36^, and cryo-EM revealed that, consistent with our mass-spectrometry data, they carry the complete set of r-proteins. The cp5S rRNA is well-resolved (Fig. 6b and Supplementary Fig. 4a), and its placement closely matches the position of the autonomous 5S rRNA in the wt ribosome (Fig. 6c).

Density for the 23S-cp5S rRNA junctions is weaker than the surrounding 5S and 23S nucleotides (Supplementary Fig. 4a), suggesting that the tethers are somewhat flexible. Nevertheless, the density was of sufficient quality to model the structure of the CUG and A linkers (at the 5′ and 3′ ends of cp5S rRNA, respectively) (Fig. 6b and Supplementary Fig. 4a). The presence of the tethers introduces only minimal perturbations in the 23S rRNA at the junction site and tethers do not clash with any of the r-proteins. Nearly all 23S and 5S rRNA residues remain at their wt position, except for the shifted G1026 and A1027 (Fig. 6d) at the site of the cp5S rRNA insertion. The tether residues form a stack with the 5′ CUG tether sandwiched between G1026 of the 23S rRNA and the 3′ A tether (Fig. 6b). Residues G1026 and A1134 of 23S rRNA are flipped by ~90° and support the tether stack (Fig. 6b, d, e). The overall structural integrity of the 23S-cp5S rRNA at the junction site as well as the repositioning of A1134 is further confirmed by chemical probing of 23S-cp5S ribosomes in solution (Supplementary Fig. 4b, c). The structural analysis clearly illustrates how the engineered hybrid 23S-cp5S rRNA can fit in the structure of functional 70S ribosomes.

### Aberrant 50S subunits have malformed PTC

In contrast to the tRNA-bound 70S ribosomes, all unassociated 50S subunits in the 70S sucrose gradient peak exhibited major structural defects (Fig. 7). The distinctive features of these particles are a drastically rearranged rRNA in the PTC (Fig. 7b, c and Supplementary Fig. 5) and lack of r-proteins uL16 and bL33 (Fig. 7d). Notably, bL35 and bL36, whose paucity was noted in proteomics studies, are stoichiometric in the 50S cryo-EM maps. It is possible that large subunits lacking these proteins formed aggregates during preparation of the sample for cryo-EM analysis (see Methods) and thus, were not visualized. Similarly misassembled 50S subunits were also found in a large fraction of 70S ribosomes that lacked tRNAs and were likely translationally inactive (Supplementary Figs. 3a and 5). Approximately half of the nucleotides of the central ring of the 23S rRNA domain V, that form the heart of the PTC, are displaced compared with their position in the wt ribosome (Fig. 7b), leading to the disruption of the network of tertiary interactions in the PTC active site^37^ (Fig. 7b, c and Supplementary Fig. 5b–d).

One of the most striking features of the distorted PTC is sequestration of the P loop. In functional ribosome, the P-loop nucleotides G2251 and G2252 that top H80 play a critical role in positioning the 3′ CCA end of the P-site tRNA for peptidyl transfer (Fig. 7c)^37,38^. In the aberrant 50S particles, the P loop-tRNA interaction is not possible because the 23S rRNA segment 2490–2505 is shifted by up to 30 Å (at C2499), forcing the P-loop residues G2251-G2252-G2253 to base-pair with C2498-C2499-U2500 (Fig. 7c). In addition, a very weak electron density of the 23S rRNA segment 2450–2456, which normally contacts P-tRNA^37^, indicates its conformational flexibility, in keeping with the functional impairment of the misassembled mutant 50S subunits. Furthermore, the observed lack of density for 7 base pairs at the bottom of stem H89 suggests disruption of the ‘double_locked_bulge/GNRA-like’ interaction between the loop of H39 and H89, which is thought to facilitate the PTC formation^39^ (Supplementary Fig. 5c). Rearrangement of the rRNA in the PTC active site and in particular partial displacement of H89 likely destabilizes the binding of the r-protein uL16 essential for placement of the tRNA in the A site (Supplementary Fig. 5e)^40,41^. Malformation of rRNA also likely prevents the timely incorporation of some other late-assembly proteins, including bL33, bL35, and bL36 (Fig. 5c). In the known intermediates of the wt 50S subunit assembly, these proteins are always underrepresented in concert with several other proteins, in particular uL10, bL12, bL28, and bL32^16^. The latter proteins, however, are present in the defective 23S-cp5S rRNA particles, suggesting that the maturation of the mutant subunits is stalled at an idiosyncratic step that represents either an unusual biogenesis intermediate or the dead-end of an off-pathway assembly route.

Cryo-EM reconstructions clearly show that depriving 5S rRNA of its autonomy dramatically derails the assembly of a large fraction of the large ribosomal subunits. The disruption of the rRNA structure and loss of uL16 would abolish correct tRNA binding in the PTC active site and prevent misassembled subunits from forming translationally-competent 70S ribosomes.

## Discussion

Our study aimed to answer two interconnected fundamental questions concerning the structure and function of the ribosome: (1) Why 5S rRNA has been preserved through the course of evolution as an individual molecule? and (2) Is there any critical cellular function that 5S rRNA can accomplish only as an autonomous molecule? The major challenge in answering these questions was that no free-living organism without free 5S rRNA had been previously found or engineered. We managed to address these questions by engineering a bacterial strain that lacks free 5S rRNA and which carries the ribosomes with 5S rRNA covalently incorporated into 23S rRNA. The existence of natural cp5S rRNAs in mitochondrial ribosomes of some algae supported the feasibility of using cp 5S rRNA in our experiments^42^.

Two (DH42 and CH84) out of the three tested constructs yielded viable cells without free 5S rRNA. This result underscores the malleability of the ribosome structure and suggests that various ribosome designs, including those lacking free 5S rRNA, have been possibly tested by nature through the course of evolution of the translation apparatus. In fact, some of the ribosomal architectures lacking 5S rRNA have been evolutionarily approved for protein synthesis in mitochondria, where 5S rRNA has been replaced by other RNAs. Specifically, in the mammalian mito-ribosomes the role of 5S rRNA has been taken over by a tRNA^43^. Furthermore, yeast mito-ribosomes feature two expansion segments of 25S rRNA that, while being structurally distinct from 5S rRNA, fill up the space in the central protuberance in place of the 5S rRNA. Notably, the expansion segments in the yeast mito-ribosomal rRNA co-localize with the sites of cp5S insertion in our successful DH42 and CH84 designs^44^. The functionality of such mitochondrial ribosomes is in line with our finding that the fully assembled engineered *E. coli* ribosomes with 23S-cp5S rRNA are nearly as active in translation as wt ribosomes, thereby reinforcing our conclusion that free 5S rRNA is not required for protein synthesis. The considerable growth rate of the DH42* cells (Fig. 3e) also argues that free 5S rRNA is not involved in extraribosomal activities critical for bacterial proliferation.

Yet, evolution has strictly preserved the autonomy of the 5S rRNA component in cytoplasmic ribosomes. The accumulation of aberrant 50S subunits in bacterial cells lacking free 5S rRNA strongly suggests that the evolutionary preservation of autonomous 5S rRNA is due to its role in ribosome assembly. The elimination of free 5S rRNA by the insertion of its sequence into 23S rRNA dramatically restricts the 5S rRNA dynamics during ribosome biogenesis and redirects the assembly of a major fraction of 50S subunits towards atypical structures. The aberrant 50S subunits likely represent dead-end products because their protein composition does not match that of the previously described on-pathway assembly intermediates of wt ribosomes^16,45^. Although large subunits with perturbed PTC were found also in a fraction of 70S ribosomes (Supplementary Figs. 3a and 5a, b), these ribosomes are translationally inactive because they are unable to bind tRNA in the P site. During normal assembly of the wt 50S subunit, free 5S rRNA could dynamically guide the large subunit biogenesis toward the functionally active structure. In particular, it may facilitate maturation of H89 and the rest of the PTC active site at late-assembly steps^39^. The critical role of the 5S rRNA dynamics in the assembly of the bacterial ribosome is generally compatible with the proposed maturation pathways of eukaryotic cytoplasmic ribosomes^46,47^. Curiously, the unwinding of H89 that we observed in the aberrant 50S subunits is reminiscent of the Nog1-mediated separation of H89 strands in the assembly intermediates of the eukaryotic large ribosomal subunit where eventual rearrangement of H89 into its mature conformation is required for uL16 binding^41,47^. In eukaryotes, Nog1 interacts with another assembly factor, Nog2^47^, which is homologous to the bacterial protein RbgA. Recent cryo-EM reconstructions of the *Bacillus subtilis* large ribosomal subunit assembly intermediates reveal local disruption of the PTC, including H89^48^ in the *rgbA* knock-out strain. Interestingly, in the wt cells, the release of RbgA and the subsequent maturation of the 50S requires the presence of uL16^49^.

Our findings provide new insights into large subunit biogenesis. Incorporation of 5S rRNA was proposed to require an extreme ~180° rotation upon its initial binding^15^. Only after such an extreme flip, 5S rRNA is thought to properly organize key functional elements of the large subunit including the central protuberance, the intersubunit bridge B1b, A-site finger (H38), H89, and PTC^15,46^. In our design, the tethering of loop D of the 5S rRNA to the 23S rRNA makes such large structural rearrangements impossible (Supplementary Fig. 6). Yet a fraction of the 23S-cp5S rRNA can assemble into fully functional ribosomes. Hence, this result is more consistent with the view that the assembly of the large ribosomal subunits can proceed via multiple pathways and that binding of the 5S rRNA in a 180° rotated state represents either one of the alternative intermediates or even a non-productive misfolding kinetic trap^16^. Further studies will be required to distinguish between these scenarios.

In conclusion, our ribosome- and cell-engineering efforts demonstrate the dispensability of the free 5S rRNA for proper protein synthesis and cell viability. Our data argue that the evolutionary preservation of the autonomous 5S rRNA is most likely due to its key role in assembly of the large ribosomal subunit where it guides the formation of the functional PTC.

## Methods

### Plasmids construction

All the plasmids were constructed by Gibson assembly^50^, with the plasmid backbones prepared by inverse PCR or restriction nuclease digest and the inserts generated by PCR or synthesized chemically by Integrated DNA Technologies. The rRNA-encoding plasmids were initially electroporated into *E. coli* POP2136 cells (genotypes of all strains used in this study are listed in Supplementary Table 1) and transformants were recovered on LB plates supplemented with the proper antibiotics; plates were incubated at 30 °C to prevent the expression of the rRNA genes controlled by the lambda P_L_ promoter^31^. All other plasmids were transformed and propagated in the *E. coli* JM109 strain and grown at 37 °C in LB media supplemented when needed with 100 μg/ml of ampicillin (Amp), 50 μg/ml of kanamycin (Kan) or 50 μg/ml of spectinomycin (Spc).

### Construction of the ptRNA100 plasmid

The plasmid backbone including the pA15 origin of replication and Spc resistance gene, was PCR amplified from the ptRNA67 plasmid^51^ using the primers NA1 and NA2 (all primers are listed in Supplementary Table 4). The tRNA gene cluster (encoding tRNA^Glu^, tRNA^Ala^, tRNA^Ile,^ tRNA^Trp^, and tRNA^Asp^), under the control of the P_tac_ promoter and T1 terminator, was synthesized as a gBlock (Integrated DNA Technology). The pTRNA100 plasmid was generated by Gibson assembly. The structure of the plasmid was verified by restriction digest and the presence of the plasmid born tRNA cluster was confirmed by PCR amplification using primers NA3 and NA4 and by sequencing.

### Construction of the pDH42, pDH39, and pCH84 plasmids

The constructs carrying hybrid 23S-cp5S rRNA gene were generated on the basis of the plasmid pAM552^29^, which harbors the *E. coli rrnB* rRNA operon. The 5S rRNA gene was deleted by inverse PCR using primers NA17 and NA18, that harbor the Xho1 restriction site, digestion of the PCR product by Xho1, and DNA ligation. The Ery resistance mutation A2058G was then introduced into the 23S rRNA gene by site-directed mutagenesis using the QuikChange Lightning Multi Site-directed mutagenesis kit (Agilent Technologies) with primer NA7. The resulting plasmid, pAM552Δ5S was used to introduce the cp5S rRNA genes with linkers at three different locations within the 23S rRNA gene.

Preparation of the cp5S rDNA sequence for integration into 23S rDNA was carried out in a single PCR reaction where primer pairs NA8/NA9 (for DH39 and DH42 constructs) or NA26 and NA27 (for CH84 construct) (100 nM each) that harbored the cp5S rDNA insert, were combined with primer pairs (250 nM each) introducing random sequence linkers, ranging in size from 0 to 3 nt, at the ‘left’ and ‘right’ 23S-cp5S junctions, as follows: NA10-NA13 and NA14-NA17 for the DH39; NA18-NA21 and NA22-NA25 for DH42; NA28-NA31 and NA32-NA35 for CH84.

For generation of the DH39, DH42, and CH84 plasmid libraries, the pAM552Δ5S plasmid was opened by inversed PCR using primer pairs NA36/NA37, NA38/NA39, and NA40/NA41, respectively. The cp5S rDNA inserts were introduced into the resulting PCR-amplified plasmid backbones by Gibson assembly reactions. The reaction products were transformed into *E. coli* POP2136 cells by electroporation and the transformants were recovered on LB/Amp agar plates grown at 30 °C (conditions that prevent expression of the plasmid-borne rRNA). Each library had at least 3-times more clones than the estimated theoretical diversity of 7225 variants. Colonies were washed off the LB plates, plasmid libraries were extracted and stored.

### Replacement of ptRNA67 with ptRNA100

The *E. coli* SQ171 strain that lacks chromosomal rRNA alleles and carries the pCSacB plasmid as the source of the rRNAs genes and ptRNA67 plasmid as the source of the missing chromosomal tRNA genes^32,52^, was used as the starting host strain. In order to replace the ptRNA67 plasmid with ptRNA100, SQ171 cells were first transformed with ptRNA-Amp (provided by Michael O’Connor, University of Missouri, Kansas City), which resembles ptRNA67 but confers Amp resistance and contains the pBR322 origin of replication. The transformants were then cured off the ptRNA67 plasmid by passaging in LB medium supplemented with Amp and Kan for ~100 generations. The loss of the ptRNA67 plasmid was verified by sensitivity of clones to Spc upon replica plating. The resulting strain was then transformed with ptRNA100 and plated on LB agar supplemented with Spc and Kan. Transformants were passaged for ~100 generations in LB media supplemented with Spc and Kan. The loss of the ptRNA-Amp plasmid was verified by sensitivity of individual clones to Amp as revealed by replica plating. The presence of ptRNA100 and the lack of ptRNA67 were additionally verified by PCR and restriction digest of the total plasmids prepared from the resulting clone.

### Inactivation of the recA gene in the SQ171/ptRNA100 cells

The *recA* gene in the SQ171/ptRNA100 strain was inactivated by P1 phage transduction. The donor strain BW25113 *recA::cat* was first prepared by the conventional recombineering procedure^52^ using chloramphenicol (Chl)-resistance cassette from the pKD3 plasmid^52^. The cassette was PCR-amplified using the primers NA42 and NA43. The PCR fragment was transformed into the BW25113 strain carrying the Red recombinase-expressing plasmid pDK46. After verification of integration of the *cat* cassette and curing off pKD46, the BW25113 *recA::cat* cells were used as the donors for the phage transduction. P1 phages transduction was carried out according to the standard protocol^53^ except that the recovery incubation was extended from 1 to 3 h before plating the transductants on LB/agar plates supplemented with Kan, Spc and 15 μg/ml Chl. For excision of the *cat* cassette, the resulting strain was transformed with pZFLP-TetR plasmid that encodes the flippase enzyme and the transformants were plated on the LB/agar plate supplemented with Kan, Spc and 2.5 μg/ml tetracycline (Tet). The elimination of the *cat* gene was confirmed by PCR and by Chl sensitivity of the clones. Then, pZFLP-TetR plasmid was cured off by passaging the cells for ~100 generations in LB medium supplemented with Kan, Spc, and the resulting clones were tested for sensitivity to Tet. The resulting strain was named SQA18 (Supplementary Table 1).

### Introduction of the 23S-cp5S libraries into SQA18 cells

The plasmid libraries extracted from POP2136 cells were transformed into SQA18 cells by electroporation. After 2 h recovery at 37 °C, transformants were plated on LB/Amp agar plates which were then incubated overnight at 37 °C. Colonies were washed off the agar plates and 0.01 A_600_ (~10^6^ cells) were placed in a volume of 2 ml of LB medium supplemented with 150 μg/mL Ery and 0.25% sucrose. After growth for 16 h, cells were plated onto agar plates containing Amp, 1 mg/ml Ery, and 5% sucrose. Plates were incubated for 48 h at 37 °C. Viable colonies appeared with DH42 and CH84 libraries, but not with the DH39 library. Several of the colonies that appeared on the plates were tested by colony PCR for the presence of 23S-cp5S rDNA using the primer pairs NA44/NA45 for the DH42 construct and primers NA46/NA47 for the CH84 construct. Absence of the wt 5S rRNA gene was verified by colony PCR using primers NA48 and NA49.

### Selection of the preferred 23S-cp5S rRNA linkers

The total DH42 plasmid library isolated from the POP2136 cells was transformed into SQA18 cells and plated on LB/Amp plates as described above. The colonies (~50,000) were washed off the plate and total plasmid was extracted from ~10^9^ cells and used as a pre-selection library.

Approximately 10^9^ cells were then subjected to the plasmid exchange procedure. Specifically, they were placed in a volume of 20 ml of LB medium supplemented with 150 μg/mL Ery and 0.25% sucrose. After growth for 4 h, cells were plated onto agar plates containing Amp, 1 mg/ml Ery, and 5% sucrose. Plates were incubated for 48 h at 37 °C. Colonies (~50,000) were washed off the plate. The extracted total plasmid from these cells represented the post-selection library.

The cp5S rDNA flanked by 23S rDNA sequences and linkers was PCR amplified from the pre-selection and post-selection library plasmid preparations using primers NA50 and NA51. The PCR libraries were subjected to next generation sequencing.

The fold-enrichment factor for each combination of linkers was calculated using the following procedure. After trimming the adapter, the entire sequence of cp5S rRNA, except for the terminal 4 nucleotides on each end, was eliminated to reduce the number of false sequence variants that appeared due to sequencing mistakes within the 5S-coding sequence. The resulting sequence motifs were counted in the pre- and post-selection libraries and the frequency of the fraction of each linker variant was calculated.

### Preparation of total cellular RNA

Overnight cultures of the *E. coli* POP2136 or SQA18 strains were grown at 30 °C or 37 °C, respectively, in LB/Amp medium. Cultures were diluted 1:100 into 2 ml of fresh medium and grown at 37 °C to A_600_ ~ 0.5. Cells were harvested by centrifugation (5000 × *g*, 1 min) and resuspended in 100 μl of lysis buffer [B-Per reagent (Thermo Scientific) supplemented with 1 mM Mg(OAc)_2_, 0.5 mM CaCl_2_, 0.1 mM EDTA, pH 8.0, 0.4 mg/ml lysozyme, 10 U/ml RQ1 RNase-free DNase (Promega)]. After subsequent addition of 400 μl of extraction buffer (50 mM Bis-Tris pH 6.5, 400 mM NaCl, 5 mM EDTA) and incubation for 5 min at room temperature, total RNA was extracted by phenol/chloroform extraction. RNA was ethanol-precipitated, the RNA pellet was washed with 1 ml of 70% ethanol, dissolved in RNase-free water, snap frozen and stored at −80 °C.

### Sucrose gradient analysis of the ribosomes and polysomes

Overnight *E. coli* cultures were diluted into 50 ml of LB/Amp medium and grown to A_600_ ~ 0.5. Chl was added to a final concentration of 125 μg/ml and after 5 min incubation, cells were harvested by centrifugation (5000 × g, 10 min, 4 °C). Cell pellets were resuspended in ice-cold lysis buffer (20 mM Tris-HCl, pH 7.5, 15 mM MgCl_2_, 1 mg/ml lysozyme, 0.25% sodium deoxycholate and 2U of RQ1 RNase-free DNase I). Cells were lysed by three cycles of freezing-thawing. The lysates were clarified by centrifugation (20,000 × g, 15 min, 4 °C) and 20 A_260_ units of lysate were loaded onto 12 mL of 10–40% linear sucrose gradient in buffer 20 mM Tris-HCl, pH 7.5, 10 mM MgCl_2_, 100 mM NH_4_Cl, 2 mM β-mercaptoethanol. Gradients were centrifuged (3 h, 4 °C) at 39,000 rpm in a SW41 rotor (Beckman). Gradients were fractionated using a fractionator (BioComp), recording the absorbance at 254 nm. Fractions corresponding to the 50S subunits, 70S ribosomes, and polysomes were pooled together. RNA was isolated by phenol/chloroform extraction and ethanol precipitation.

### Isolation of 70S ribosomes and ribosomal subunits

For isolation of tight-couple ribosomes^54^, overnight *E. coli* cultures were diluted into 1 L of LB/Amp medium and grown to A_600_ ~ 0.5. Cells were harvested by centrifugation (5,000 g, 15 min, 4 °C), resuspended in buffer A (20 mM Tris-HCl, pH 7.5, 100 mM NH_4_Cl, 10 mM MgCl_2_, 0.5 mM EDTA, pH 8.0, 6 mM β-mercaptoethanol, 10 U/ml of RQ1 RNase-free DNase I), and lysed using French press at 10,000 psi. Lysates were clarified by centrifugation in JA25–50 rotor (Beckman) at 13,000 rpm for 30 min at 4 °C and supernatants were loaded onto 10 ml 1.1 M sucrose cushion prepared in a buffer containing 20 mM Tris-HCl, pH 7.5, 500 mM NH_4_Cl, 10 mM MgCl_2_, 0.5 mM EDTA, pH 8.0 and 6 mM β-mercaptoethanol, in 35 ml Quick-Seal centrifuge tubes (Beckman). Ribosomes were pelleted by centrifugation for 16 h at 36,000 rpm (4 °C) in a Ti70 rotor (Beckman). The ribosome pellet was resuspended in buffer B (20 mM Tris-HCl, pH 7.0, 6 mM MgCl_2_, 100 mM NH_4_Cl and 6 mM β-mercaptoethanol) and 100 A_260_ units were loaded onto 10–40% sucrose gradient prepared in buffer B in the tubes of a SW27 rotor (Beckman). Gradients were centrifuged for 21 h at 20,000 rpm (4 °C) in a SW27 rotor, fractionated using gradient fractionator (BioComp) and fractions containing 70S ribosomes and 50S ribosomal subunits were pooled separately. Material was concentrated using 2 ml Vivaspin concentrators (Sartorius) with cellulose triacetate membrane and recovered in ribosome storage buffer (20 mM Tris-HCl, pH 7.0, 10 mM MgCl_2_, 100 mM NH_4_Cl, 6 mM β-mercaptoethanol). Aliquots of ribosomes and ribosomal subunits were flash-frozen and stored at −80 °C. When needed, rRNA was isolated by phenol/chloroform extraction and ethanol precipitation.

For isolation of the 50S subunits from the 70S ribosomes, 130 pmol ribosomes, prepared as described above but concentrated by pelleting and stored in the ribosome storage buffer (20 mM Tris-HCl, pH 7.0, 10 mM MgCl_2_, 100 mM NH_4_Cl, 6 mM β-mercaptoethanol) were diluted in buffer D (20 mM Tris-HCl, pH 7.5, 100 mM NH_4_Cl, 0.5 mM EDTA, 6 mM β-mercaptoethanol) to reach a 1.5 mM final concentration of MgCl_2_. Ribosomal subunits were separated by centrifugation in 10–40% sucrose gradients prepared in buffer D that contained 1.5 mM MgCl_2_. Gradients were centrifugated for 16 h at 27,000 rpm (4 °C) in a SW27 rotor (Beckman), fractionated, concentrated, and recovered in the ribosome storage buffer, as described above. Aliquots were flash-frozen and stored at −80 °C.

### LC-MS/MS analysis of ribosomal proteins

Protein composition of wt and mutant tight-couple 70S ribosomes and 50S ribosomal subunits, prepared as described above, was determined using quantitative mass-spectrometry^55^. Ribosomal preparations were mixed in a 1:1 molar ratio with SILAC-labeled wt ribosomes containing mass-labeled arginine (Arg6: [^13^C]_6_HN_4_O_2_ and lysine (Lys4: C_6_H_10_[^2^H]_4_N_2_O_2_) (Silantes GmbH, Germany). Ribosomal proteins were digested with trypsin (Sigma) or Lys-C protease (Wako, USA). Resulting peptides were fractionated and analyzed via LC-MS/MS using LTQ-Orbitrap XL (Thermo Scientific). Protein identification and quantification analysis were performed using Maxquant v1.5.6.0^56^ and Perseus v1.6.2.3^57^. Maxquant search was done using *E. coli* MG1655 protein sequence database from UniProtKB (24.04.2019). Protein abundance was normalized for large and small subunit proteins separately by shifting mean L/M ratio to 1.

### Chemical probing of rRNA structure

rRNA structure was probed in the 70S ribosomes isolated from wt or mutant cells. The ribosomes (10 pmol) were placed in 50 μl of modification buffer [80 mM HEPES-KOH, pH 7.6, 15 mM MgCl_2_, 100 mM NH_4_Cl containing 20 U of RiboLock RI RNase inhibitor (Thermo Scientific)] and activated by incubation for 5 min at 42 °C. The modification reaction was started by addition of 2 μl of dimethyl sulfate (Sigma) diluted 1:10 in ethanol. Samples were incubated for 10 min at 37 °C. The modification reactions were stopped by addition of 50 μl of freshly prepared stop solution (600 mM NaOAc, 1 M β-mercaptoethanol) and 300 μl of ethanol. Samples were precipitated, resuspended in buffer (300 mM sodium acetate, 5 mM EDTA, pH 8.0, pH 7.0, 0.5% SDS) and RNA was isolated by phenol/chloroform extraction and ethanol precipitation. Primer extensions were carried out using primers NA56-NA57.

### Analysis of the mutant rRNA content

For the analysis of the presence of the engineered 23S-cp5S rRNA, total RNA was isolated from the sucrose gradient fractions as describes above. The mutant 23S-cp5S rRNA content was assessed by primer extension using either NA58 or NA59 primers. Specifically, 5′-[^32^P]-labeled primer (0.5 pmol) was annealed to 1 μg of total RNA in hybridization buffer (50 mM K-HEPES, pH 7.0, 100 mM KCl) by incubating at 90 °C for 1 min and then cooling over 15 min to 42 °C, and extended with 2 U of AMV reverse transcriptase (Roche) for 20 min at 42 °C in a final reaction volume of 8 µl. For the NA58 primer, the reaction contained 0.25 mM of ddATP and 0.2 mM of dCTP, dGTP, dTTP. For the NA59 primer, the reaction contained 0.25 mM of ddCTP and 0.2 mM of dATP, dGTP, dTTP. Reactions were terminated by adding 120 μl of stop buffer (84 mM NaOAc, 0.8 mM EDTA, pH 8.0, 70% EtOH), cooling at −80 °C for 15 min, and pelleting nucleic acids by centrifugation at 15,000 × *g* for 1 h at 4 °C. Supernatants were removed, pellets were dried and dissolved in formamide loading dye. The cDNA products were resolved in a 12% denaturing polyacrylamide gel and visualized by phosphorimaging.

### In vitro translation

In vitro translation reactions were carried out in the Δribosome PURExpress cell-free translation system (New England Biolabs). The DNA templates containing the T7 RNA polymerase promoter, ribosome binding site, and the protein-coding sequence were prepared by PCR. The *ermBL* template was prepared using primers NA52-NA55. The GFP template was generated from the *sf-gfp* gene of plasmid pY71-sfGFP^58^ using primers NA31 and NA32. Dihydrofolate reductase (DHFR) was expressed from the plasmid template supplied with the PURExpress kit.

Translation reaction for expression of GFP was assembled in a total volume of 10 μl and contained 2 μl of the PURExpress kit solution A, 1.2 μl of factor mixture, 1 μl amino acid mixture (3 mM each), 1 μl tRNA (20 μg/ml), 16 U of RiboLock RNase inhibitor (ThermoFisher), 10 ng GFP template and 12 pmol of wt or mutant ribosomes. Samples were placed in wells of a 384-well black wall/clear flat bottom tissue-culture plate (BD Biosciences) and covered with the lid. Reactions were incubated at 37 °C in a microplate reader (Tecan) with GFP fluorescence being recorded every 10 min at *λ*_exc_ = 488 nm and *λ*_em_ = 520 nm over a period of 4 h.

Expression of DHFR was followed by incorporation of [^35^S]-L-methionine into the full-size protein. Translation was carried out in 10 μl reactions assembled as described above but supplemented with 5 μCi [^35^S]-L-methionine (1175 Ci/mmol), using 50 ng of the DNA template and 6 pmol of wt or mutant ribosomes. Reactions were incubated at 37 °C. Every 12 min, 1 μl aliquots were withdrawn, mixed with 3 μl of SDS-containing gel loading dye and stored on ice until the reaction course was completed. The protein products were analyzed by SDS–gel electrophoresis in 16.5% Bis-Tris gels (Biorad) using NuPAGE MES/SDS running buffer (Invitrogen). Gels were stained, dried, and exposed to a phosphorimager screen overnight. Radioactive bands were visualized by phosphorimaging.

### Cryo-EM analysis of the 70S ribosomes and 50S subunits

Cryo-EM grids were prepared as follows. 400 M copper grids coated with lacey carbon and a 2 nm thin layer of carbon (Ted Pella Inc.) were glow discharged with 20 mA current with negative polarity for 60S in a PELCO easiGlow glow discharge unit. A Vitrobot Mark IV (ThermoFisher) was pre-equilibrated to 4 °C and 95% relative humidity. An aliquot of previously flash-frozen ribosomes was thawed and when opalescence was noted, the solution was cleared in a micro centrifuge (10 minutes at 16,000 × *g* at 4 °C). The concentration of the cleared supernatant was derived from A_260_ measured using a Nanodrop (ThermoFisher) and the ribosomes were diluted to 200 nM in LPP Buffer [10 mM Tris-HCl, pH 7.0, 60 mM KCl, 60 mM NH_4_Cl, 12 mM Mg(CH_3_COO)_2_]. Three µl of 200 nM ribosome solution were applied to grid; after a 10S delay the grid was blotted for 4S and then plunged into liquid-nitrogen-cooled liquid ethane.

Data were collected on a Talos electron microscope (ThermoFisher) operating at 200 KV and equipped with a K3 direct electron detector (Gatan Inc.) targeting 0.5–1.8 μm underfocus. Data collection was automated with SerialEM^59^ using beam tilt to collect multiple movies (e.g. one shot on center, 6 with shift) at each stage position^60^. Super-resolution movies had a total of 19 frames with 1.5 e^−^/Å^2^ per frame for a total dose of 30 e^−^/Å^2^ on the sample. A total of 5222 movies were collected over 22 h. Movies were aligned on the fly during data collection using IMOD^61^ to decompress frames, apply the gain reference, bin the super-resolution data to physical pixel of 0.87 Å on sample, and correct for image drift.

Early steps of 3D map generation from CTF determination, reference-free particle picking (489,732 particles), and stack creation were carried out in cisTEM. Particle alignment and refinement were carried out in Frealign 9.11 and cisTEM^62,63^. To speed up processing, 2×-, 4×-, and 8×-binned image stacks were prepared using resample.exe, which is part of the FREALIGN distribution^64^. The initial model for particle alignment of 70S maps was EMD-1003^65^, which was downsampled to match 8× binned image stack using EMAN2^66^. Two rounds of mode 3 search with a high-resolution cutoff of 30 Å then 20 Å were run using the 8× binned stack. Next, 7 rounds of mode 1 refinement were run with the 4×, eventually unbinned stack as resolution shells were gradually added (limit of 5 Å) and resolution reached 2.94 Å. Beam shift refinement was used to improve the overall resolution to 2.87 Å.

Twenty rounds of 3D maximum-likelihood classification without masking and to 14 Å resolution was used to rapidly separate 8x binned particles into 12 classes of different compositions revealing 50S subunits, 70S ribosomes without tRNA, or 70S ribosomes with tRNAs and the 30S in either a rotated or non-rotated conformation (Supplementary Fig. 3a). 50S particle (133,490), empty 70S particles (120,428), or tRNA-bound, unrotated state 70S particle (55,677) substacks were created using the 1x binned stack using merge_classes.exe, part of the Frealign package, requiring particle scores of 0 and minimum of 50% occupancy. The substacks were then again binned to 4x using resample.exe to speed further classification steps.

The 50S particle substack was separated into 6 classes with a 55-Å radius focus mask around the 5S rRNA portion of the 23S-cp5S hybrid rRNA. Classes with weak or absent uL16 and/or bL33 and classes differing in 5S rRNA position were separated. The classes were reconstructed with original alignment and beam tilt parameters at 1x binning and one of these classes was used for structural modeling as detailed in the following section.

The 70S particles were also investigated. The empty (no tRNA) 70S particle substack was separated into 12 classes using the same 55-Å focus mask. The classes exhibited differences in uL16 and/or bL33 occupancy and 5S rRNA position, similar to the 50S classes described above. However, in contrast to the 50S particles, 3 out of 12 empty 70S classes revealed a normally folded PTC.

Classification of the classical-state tRNA-bound 70S particle substack into 12 classes with the same mask revealed nearly stoichiometric occupancy of 16 uL, and all particles had a normally folded PTC and strong P-tRNA density. In contrast, the tRNA occupancy of the A site was weak in some classes with weaker L33 density. Classification of the 70S particles with a rotated 30S and hybrid-state P/E-tRNA into 12 classes also revealed varying occupancies of uL16 and bL33, and seven classes featured aberrant PTC.

The 3.2-Å cryo-EM structure of the 70S ribosome bound with the A and P tRNAs^67^ was used as a starting model for structure refinements. The ribosome components/domains were fitted into maps using Chimera^68^. Here, the 50S, L1 stalk, L11 stalk, 30S body and head, and tRNAs were fitted independently. Modeling of 5S rRNA, and adjustments to the PTC and decoding center were made using PyMOL^69^. The structural models were refined using real-space simulated-annealing refinement using RS Ref ^70,71^ against corresponding maps. Refinement parameters, such as the relative weighting of stereochemical restraints and experimental energy term, were optimized to produce the optimal structure stereochemistry, real-space correlation coefficient, and R-factor, which reports on the fit of the model to the map^72^. Secondary structure restraints, comprising hydrogen-bonding restraints for ribosomal proteins and base-pairing restraints for RNA molecules were employed as described^73^. The structures were next refined using phenix.real_space_refine^74^ followed by a round of refinement in RSRef applying harmonic restraints to preserve protein geometry^70,71^. Phenix was used to refine B-factors of the models against their respective maps without B-factor filtering^74^. The resulting structural models have good stereochemical parameters, characterized by low deviation from ideal bond lengths and angles and agree closely with the corresponding maps as indicated by high correlation coefficients and low real-space R factors (Supplementary Table 2). Figures were prepared in PyMOL^69^.

### Reporting summary

Further information on research design is available in the Nature Research Reporting Summary linked to this article.

## Supplementary information

Supplementary Information

Peer Review File

Reporting Summary

## Data Availability

The data that support the findings of this study are available from the corresponding author upon reasonable request. The models generated and analyzed during the current study will be available from the RCSB Protein Data Bank: PDB 6WNW [10.2210/pdb<6WNW>/pdb] (unperturbed 70S ribosome), PDB 6WNT [10.2210/pdb<6WNT>/pdb] (perturbed 50S subunit), PDB 6WNV [10.2210/pdb<6WNV>/pdb] (70S ribosome with the perturbed 50S subunit). The cryo-EM maps used to generate models will be available from the Electron Microscopy Database: EMD-21858 (unperturbed 70S ribosome), EMD-21856 (perturbed 50S subunit), EMD-21857 (70S ribosome with the perturbed 50S subunit). Proteomics data can be found in the EMBL-EBI Proteomics IDEntification database (PRIDE) under accession code PXD019058. The source data underlying Figs. 2f, 3a–c, 5c and Supplementary Figs 2 and 4c, d), including the uncropped gels (Figs. 2f, 3a–c, Supplementary Fig. 4c, d, and bar graph numeric data (Fig. 5c and Supplementary Fig. 2) are provided in the Source Data file. Source data are provided with this paper.

## Source data

Source Data
