# Supplementary material for: Ribosome engineering reveals the importance of 5S rRNA autonomy for ribosome assembly
Source: Nat Commun. 2020 Jun 9;11:2900. doi: 10.1038/s41467-020-16694-8 (PMC7283268; doi:10.1038/s41467-020-16694-8)
Supplement: Supplementary file 1 — Supplementary Information [file 41467_2020_16694_MOESM1_ESM.pdf]

SUPPLEMENTARY INFORMATION

Ribosome engineering reveals the importance of 5S rRNA autonomy for ribosome assembly

Huang et al.

Supplementary Table 1. Genotypes of *E. coli* strains used in the study

Strain	Genotype	Reference
SQA18	*ilvG rfb-50 rph-1 ΔrrnGADEHBC DEL(recA)/ ptRNA100, pA15, SpcR, alaT, aspT, ileT, trpT, gltT / pH42, pBR322 ori, AmpR, 23S-5S genes phusion*	[1,2]
SQ171	*ilvG rfb-50 rph-1 ΔrrnGADEHBC / ptRNA67, pA15, SpcR, alaT, aspT, ileT, trpT, gltT*	[1,2]
JM109	*F´ traD36 proA+B+ lacIq Δ(lacZ)M15/ Δ(lac-proAB) glnV44 e14- gyrA96 recA1 relA1 endA1 thi hsdR17*	[3]
POP2136	*F- supE44 hsdR17 mcrA+ mcrB+ endA1 thi-1 aroB mal- cI857 lambda PR tetR lambda*	[4]
BW25113	Δ(*araD-araB*)*567* Δ(*rhaD-rhaB*)*568 ΔlacZ4787* (::rrnB-3) *hsdR514 rph-1*	[5]

Supplementary Table 2. Cryo-EM data collection, refinement and validation statistics

	50S	Empty 70S	70S with A- & P-tRNA
Data collection and processing			
Magnification	57,471x	57,471x	57,471x
Voltage (kV)	200	200	200
Electron exposure (e–/Å2)	30	30	30
Defocus range (μm)	0.4-3	0.4-3	0.4-3
Pixel size (Å)	0.87	0.87	0.87
Symmetry imposed	C1	C1	C1
Initial particle images (no.)	489,732	489,732	489,732
Final particle images (no.)	21,705	9,620	26,717
Map resolution (Å)**	3.1	3.5	3.2
FSC threshold	0.143	0.143	0.143
Map resolution range (Å)	2.6 - >8	3.0 - >8	2.7 - >8
Refinement			
Initial model used (PDB code)	5UYM	5UYM	5UYM
Correlation Coefficient (cc_mask)*	0.85	0.83	0.83
Real-space R-factor †	0.21	0.21	0.20
Map-sharpening B factor (Å2)	0	-25	-50
Model composition*			
Non-hydrogen atoms	91,296	143,188	148,080
Protein residues	3,387	5,782	6,089
RNA residues	3,031	4,570	4,733
B factors (Å2)*			
Protein	168.5	179.4	113.8
RNA	150.6	160.3	103.9
R.m.s. deviations*,§			
Bond lengths (Å)	0.007	0.007	0.007
Bond angles (°)	0.9	1.0	1.0
Validation*			
MolProbity score	2.21	2.25	2.18
Clashscore	12.6	12.4	10.7
Poor rotamers (%)	0.8	0.8	0.8
Ramachandran plot*			
Favored (%)	88.1	85.9	86.3
Allowed (%)	11.2	12.6	13.0
Disallowed (%)	0.7	1.5	0.7
Validation (RNA)*			
Good sugar pucker (%)	99.7	99.7	99.7
Good backbone (%)#	87.3	84.9	85.9

** from Frealign (FSC_part)
* from Phenix
† from RSRef
§ root mean square deviations
# RNA backbone suites that fall into recognized rotamer conformations defined by Molprobity

Supplementary Table 3. Structural classes found during cryo-EM classification

50S subunits (133.490 particles)					
**Class**	**% of particles**	**Resolution, Å**	**PTC state**	**Presence of uL16**	**Presence of bL33**
1[a)]	16.3	3.1	Perturbed	-	-
2	21.9	3.1	Perturbed	-	-
3	8.8	3.2	Perturbed	-	-
4	18.3	3.1	Perturbed	-	+
5	19.3	3.1	Perturbed	+	+
6	15.4	3.2	Mixed	+	+
**70S without tRNAs (120.428 particles)**					
1[a)]	8.0	3.5	Perturbed	-	-
2	12.4	3.4	Perturbed	-	-
3	7.0	3.6	Perturbed	-	+
4	5.6	3.6	Perturbed	+	-
5	8.4	3.5	Perturbed	+	+
6	5.6	3.6	Perturbed	+	+
7	7.0	3.5	Perturbed	+	+
8	8.1	3.6	Perturbed	+	+
9	6.6	3.5	Perturbed	+	+
10	16.8	3.3	Normal	+	+
11	7.5	3.5	Normal	+	+
12	6.8	3.5	Normal	+	+
**Rotated 70S with tRNAs (101.779 particles)**					
1	9.3	3.7	Perturbed	-	+
2	8.4	3.7	Perturbed	-	+
3	8.0	3.7	Perturbed	-	+
4	5.8	3.9	Perturbed	+	+
5	7.8	3.7	Perturbed	+	+
6	6.6	3.8	Perturbed	+	+
7	5.7	3.9	Perturbed	+	+
8	13.0	3.5	Normal	+	+
9	9.9	3.6	Normal	+	+
10	8.0	3.7	Normal	+	+
11	9.3	3.7	Normal	+	+
12	8.1	3.7	Normal	+	+
**Non-rotated 70S with tRNAs (55.677 particles)[b)]**					
**Class**	**% of particles**	**Resolution, Å**	**A-site tRNA**	**P-site tRNA**	**E-site tRNA**
1[c)]	8.1	3.8	+	+	±[d)]
2[c)]	9.0	3.9	+	+	-
3[c)]	9.3	3.8	+	+	-
4[c)]	8.5	3.8	+	+	-
5[c)]	7.5	3.9	+	+	-
6[c)]	7.3	3.9	+	+	-
7	6.6	3.9	±[d)]	+	-
8	9.0	3.8	-	+	-
9	8.4	3.8	-	+	-
10	11.2	3.7	-	+	-
11	7.0	4.0	-	+	-
12	8.1	3.8	-	+	±[d)]

[a)] The map of this class was used for modeling

[b)] All ribosomes in non-rotated state have uL16 and bL33 bound. The conformation of PTC is normal in all classes.

[c)] These classes were merged to prepare the 3.2-Å resolution map used for modeling the non-rotated 70S with tRNAs

[d)] Weak density

Supplementary Table 4. Primers used in the study

Primer	Sequence
NA1	GAAGACTGGGCCTTTTGTTTTATGTCGACGGGCATAAATAGGTTTAATTTTGCTACG
NA2	CAATATTTTTTTGATGGAAAGGAAAGAGATCTCGGATCCTCGCTAGATTTTAATGCGG
NA3	TTAAGCGTGCATAATAAGCCC
NA4	CGAATTCTTTTACAACCATCG
NA5	GGCTCGAGGCATCAAATAAAACGAAAGGCTC
NA6	GGCTCGAGGTTTTACTGCTCATTTTCAT
NA7	CGCGGCAAGACGGGAAGACCCCGTGAA
NA8	TCCCCATGCGAGAGTAGGGAACTGCCAGGCCTTCGGGCCTGGCGGCAGTAGCGCGGTG GTCCCACCTGACCCCATGCCGAAC
NA9	GACCCCACACTACCATCGGCGCTACGGCGTTTCACTTCTGAGTTCGGCATGGGGTCAGG TGGGACCA
NA10	TCACGGGAGACACACGGCGGGTGCTTCCCCATGCGAGAGTAGGGA
NA11	TCACGGGAGACACACGGCGGGTGCTNTCCCCATGCGAGAGTAGGGA
NA12	TCACGGGAGACACACGGCGGGTGCTNNTCCCCATGCGAGAGTAGGGA
NA13	TCACGGGAGACACACGGCGGGTGCTNNNTCCCCATGCGAGAGTAGGGA
NA14	TGTTTCCCTCTTCACGACGGACGTTGACCCCACACTACCATCGGC
NA15	TGTTTCCCTCTTCACGACGGACGTTNGACCCCACACTACCATCGGC
NA16	TGTTTCCCTCTTCACGACGGACGTTNNGACCCCACACTACCATCGGC
NA17	TGTTTCCCTCTTCACGACGGACGTTNNNGACCCCACACTACCATCGGC
NA18	GGTCCCAAAGTCATGGTTAAGTGGGCTCCCCATGCGAGAGTAGG
NA19	GGTCCCAAAGTCATGGTTAAGTGGGNCTCCCCATGCGAGAGTAGG
NA20	GGTCCCAAAGTCATGGTTAAGTGGGNNCTCCCCATGCGAGAGTAGG
NA21	GGTCCCAAAGTCATGGTTAAGTGGGNNNCTCCCCATGCGAGAGTAGG
NA22	GTCTGGGCCTTCCCACATCGTTTACCCCACACTACCATCGGC
NA23	GTCTGGGCCTTCCCACATCGTTTNACCCCACACTACCATCGGC
NA24	GTCTGGGCCTTCCCACATCGTTTNNACCCCACACTACCATCGGC
NA25	GTCTGGGCCTTCCCACATCGTTTNNNACCCCACACTACCATCGGC
NA26	CCGAACTCAGAAGTGAAACGCCGTAGCGCCGATGGTAGTGTGGGGTCTCCCCATGCGA GAGTAGGGAACTGCCAGGCATTCGTG
NA27	CATGGGGTCAGGTGGGACCACCGCGCTACTGCCGCCAGGCACGAATGCCTGGCAGTTC CC
NA28	GAAGGTTGGCTAATCCTGGTCGGACCCGAACTCAGAAGTGAAACGCC
NA29	GAAGGTTGGCTAATCCTGGTCGGACNCCGAACTCAGAAGTGAAACGCC
NA30	GAAGGTTGGCTAATCCTGGTCGGACNNCCGAACTCAGAAGTGAAACGCC
NA31	GAAGGTTGGCTAATCCTGGTCGGACNNNCCGAACTCAGAAGTGAAACGCC
NA32	GCCATTGCACTAACCTCCTGATCATGGGGTCAGGTGGGAC
NA33	GCCATTGCACTAACCTCCTGATNCATGGGGTCAGGTGGGAC
NA34	GCCATTGCACTAACCTCCTGATNNCATGGGGTCAGGTGGGAC
NA35	GCCATTGCACTAACCTCCTGATNNNCATGGGGTCAGGTGGGAC
NA36	AGCACCCGCCGTGTGTCTCC
NA37	AACGTCCGTCGTGAAGAGGGA
NA38	AAACGATGTGGGAAGGCCCAGAC

NA39	CCCACTTAACCATGACTTTGGG
NA40	ATCAGGAGGTTAGTGCAATGGC
NA41	GTCCGACCAGGATTAGCCAACCTTC
NA42	CAGAACATATTGACTATCCGGTATTACCCGGCATGACAGGAGTAAAAATGGTGTAGGCTG GAGCTGCTTC
NA43	ATGCGACCCTTGTGTATCAAACAAGACGATTAAAAATCTTCGTTAGTTTCATGGGAATTAG CCATGGTCC
NA44	GACTAATGTTGAAAAATTAGC
NA45	GTCGCTGCCGCAGCTTC
NA46	GCTTGACACTGAACATTGAGCC
NA47	CCAAACACCGCCGTCGATATG
NA48	TGCCTGGCGGCAGTAGCGCG
NA49	ATGCCTG GCAGTTCCTACTC
NA50	ACACTGACGACATGGTTCTACAAGGTCCCAAAGTCATGGTTAAG
NA51	TACGGTAGCAGAGACTTGGTCTGTCTGGGCCTTCCCACA
NA52	TAATACGACTCACTATAGGGCTTAAGTATAAGGAGGAAAAAATATGTTGGTATTCCAAATG CGTAATGTAGATAAAACATCTAC
NA53	GGTTATAATGAATTTTGCTTATTAACGATAGAATTCTATCACTTATTTCAAAATAGTAGATG TTTTATCTACATTACG
NA54	TAATACGACTCACTATAGGG
NA55	GGTTATAATGAATTTTGCTTATTAAC
NA56	ATGCCTGGCAGTTCCTACTC
NA57	GGCTGCTTCTAAG
NA58	TCACGACGGACGTTAGCA
NA59	GTAAAGGTTCACGGGGTC
NV1	GGTTATAATGAATTTTGCTTATTAAC

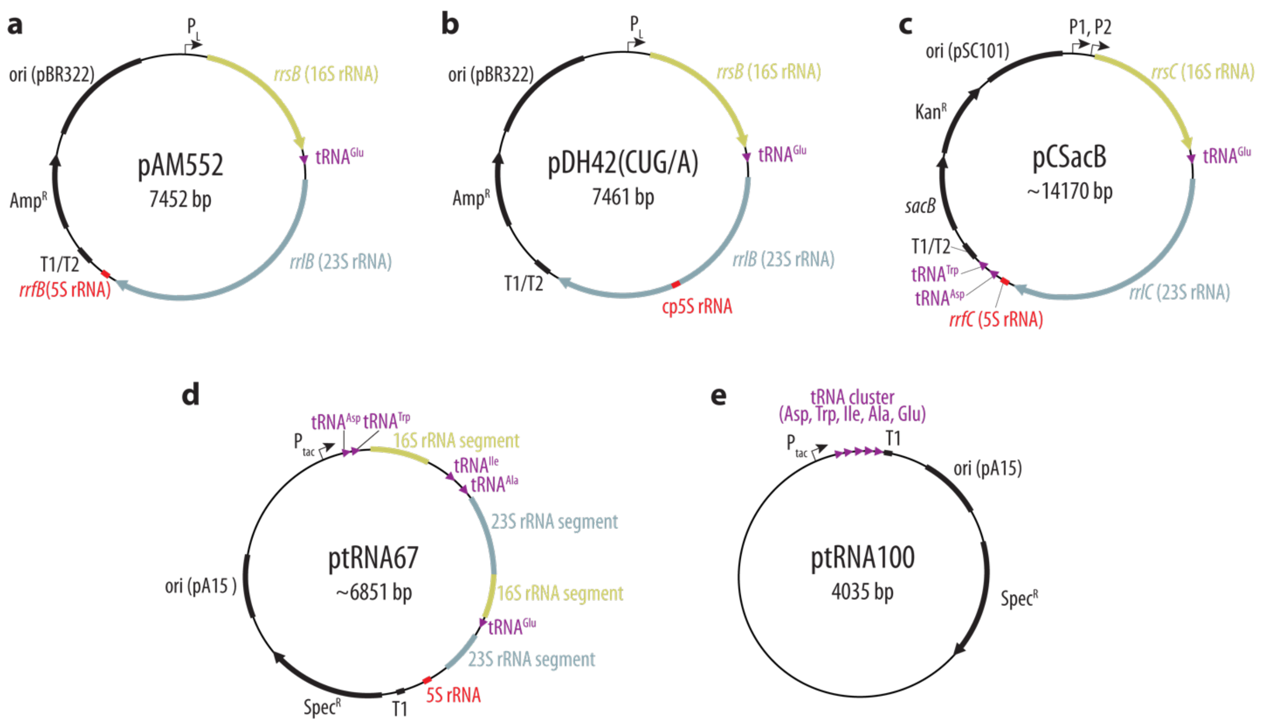

Supplementary Fig. 1 | The plasmids used in this study. a The pAM552 plasmid[2] contains the *E. coli rrnB* operon under the control of the lambda phage P_L promoter. The genes of 16S rRNA, 23S rRNA, 5S rRNA, and tRNA^{Glu} are indicated. The plasmid contains the pBR322 origin of replication and ampicillin resistance gene (Amp^r). **b** The map of the pDH42 plasmid derived from pAM552. The native 5S rRNA gene was deleted and the cp5S rRNA sequence was inserted in the 23S rRNA gene via the CUG/A tethers. **c.** pCSacB plasmid[1] contains the *E. coli rrnC* operon transcribed from its native P1 and P2 promoters. pCSacB contains the pSC101 origin of replication, kanamycin resistance gene (Kan^R) and the counter-selectable *sacB* gene restricting the ability of cells to grow on media supplemented with sucrose. **d** The ptRNA67 plasmid[6] that re-introduces missing tRNA genes into the original SQ171 strain[1]. The wt 5S rRNA and fragments of 16S and 23S rRNA genes present in the plasmid are shown. ptRNA67 carries the p15A origin of replication and a spectinomycin resistance gene (Spc^R). **e** The streamlined ptRNA100 plasmid that contains the synthetic cluster of the tRNA genes missing in SQ171 strain. The fully annotated complete sequences of the pAM552, pDH42 and ptRNA100 plasmids can be found in the Supplementary Information File.

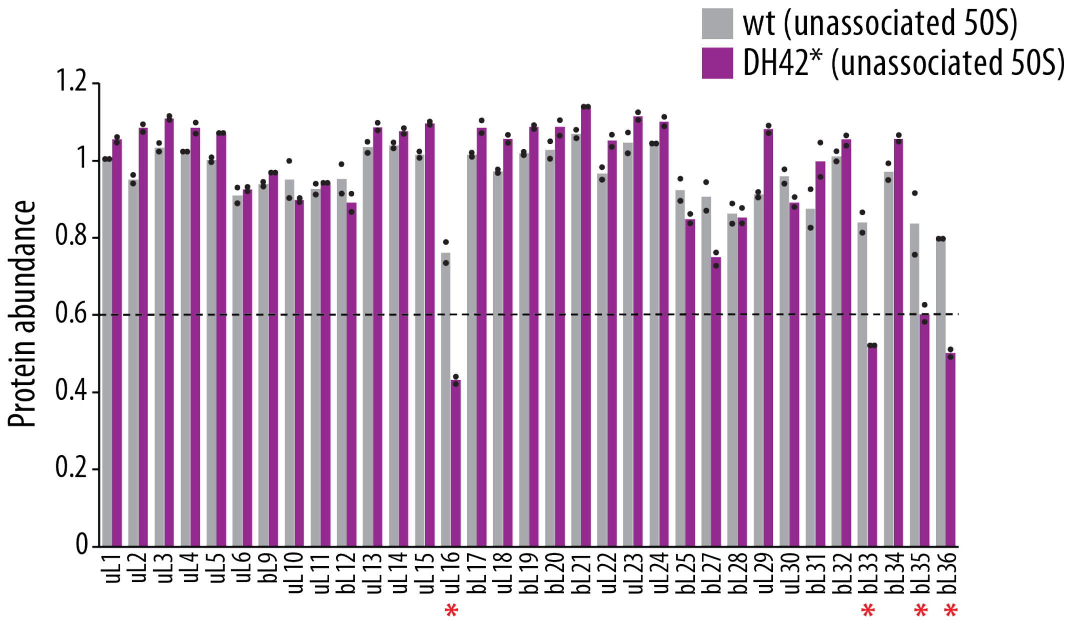

Supplementary Fig. 2 | Quantitative proteomics analysis of individual r-proteins in the unassociated wt or 23S-cp5S large ribosomal subunits. Comparison of the r-protein composition of the 50S peak from the sucrose gradient fractionation of the ribosomal material isolated from cells with wt ribosomes or expressing the mutant DH42* ribosomes. Red asterisks indicate r-proteins that are significantly underrepresented in the unassociated 50S subunits of the 23S-cp5S ribosome. The bar graph represents the mean of two biological replicates with individual data points indicated by black dots. The raw data can be found in the Source data file.

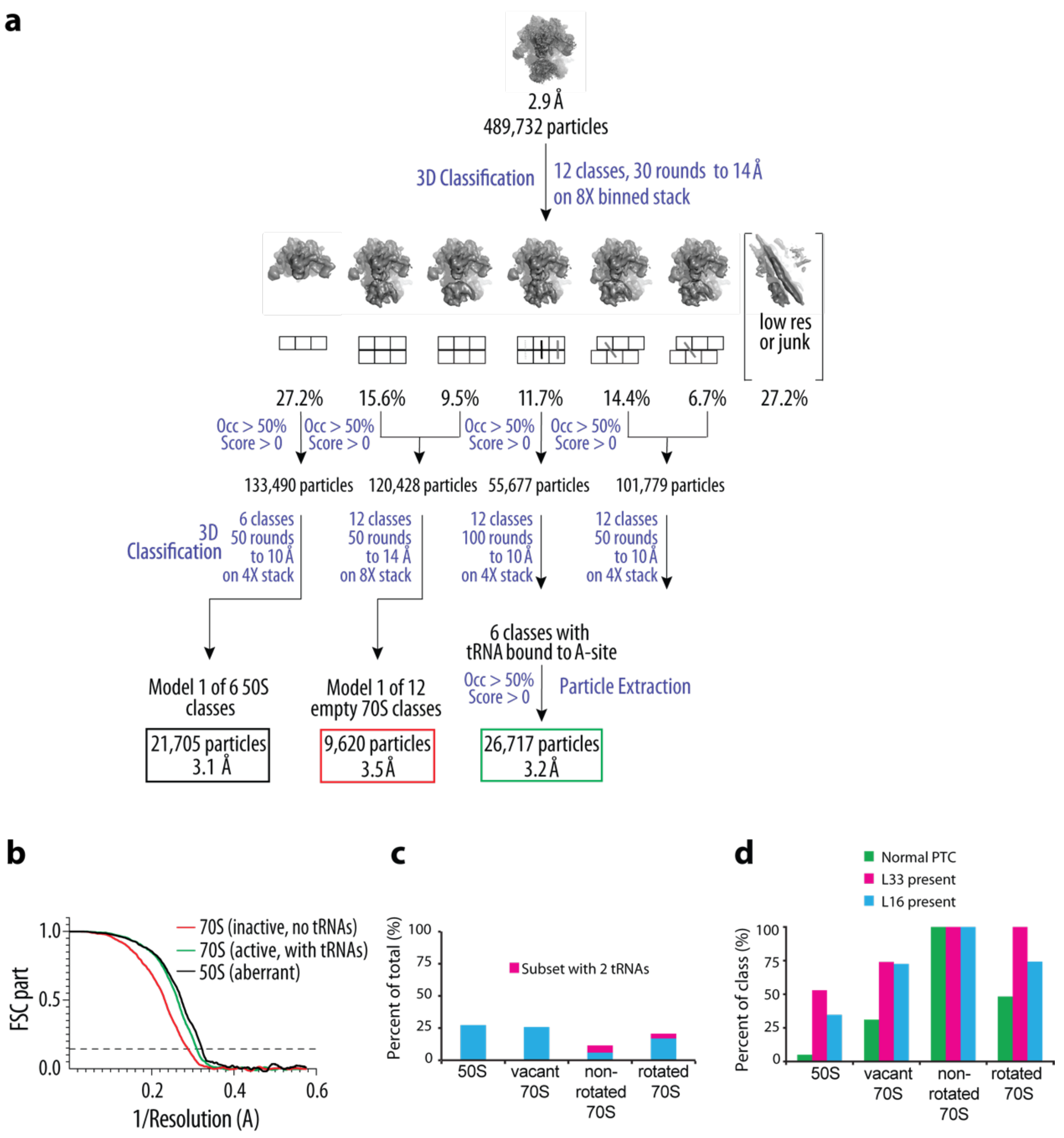

Supplementary Fig. 3 | a Scheme of cryo-EM data classification. All six resulting 50S maps contained aberrant PTC conformation, and the most resolved map was used for structural modeling (black box). Nine classes (out of twelve) of empty 70S particles had the aberrant PTC, one of them was used for structural modeling (red box). All twelve classes of the non-rotated 70S particles bound with P-tRNA had a normally folded PTC and were bound stoichiometrically with L16 and L33. A second classification step was used to separate particles bound with both P-and A-tRNAs, resulting in the map used for structural modeling (green box). **b** Fourier shell correlation (FSC) between even- and odd-particle half maps (black) show that map resolutions range from 3.1 to 3.5 Å for the 50S and 70S classes used for structural modeling (at FSC = 0.143, dashed line). **c** Distribution of particles among major ribosome states as derived from extracted particle stacks. **d** Percentage of particles with normally folded peptidyl transferase center, bL33 or uL16 protein occupancy for each of the major ribosome states.

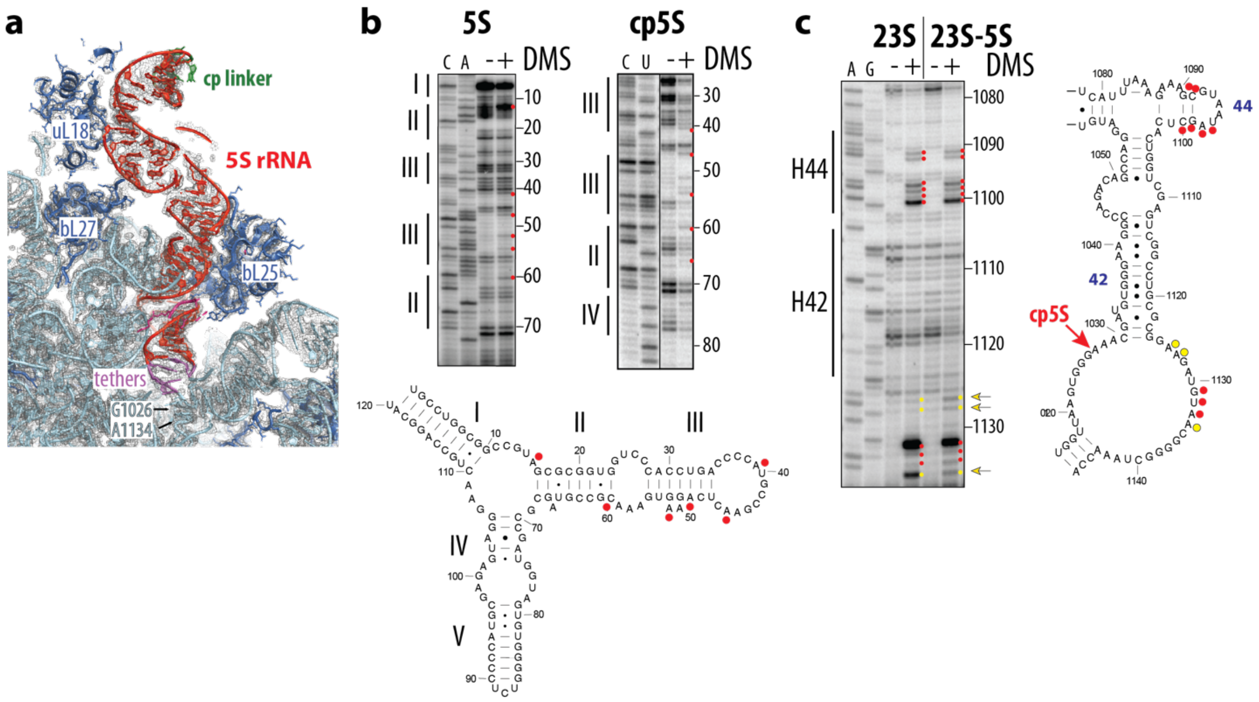

Supplementary Fig. 4 | The structure of the 5S rRNA and 23S rRNA are only minimally perturbed in the hybrid 23S-cp5S rRNA ribosomes. a Cryo-EM density (mesh) showing 23S-cp5S rRNA (light blue and red) and ribosomal proteins (dark blue) in the vicinity of the 23S and cp5S rRNA junction at σ = 1.9. **b** Chemical probing using dimethylsufate modification of 5S rRNA and cp5S rRNA structures in wt and DH42* 70S ribosomes, respectively. The 5S rRNA stems are indicated with Roman numerals at the secondary structure diagram and next to the gels. **c** Dimethylsulfate probing of the 23S rRNA structure in wt and DH42* ribosomes in the vicinity of the 23S-cp5S rRNA junction. The 23S rRNA hairpins 42 and H44 are marked. The site of the cp5S rRNA insertion is indicated b an arrow. In **b** and **c** the rRNA residues showing similar accessibility to DMS in wt and 23S-cp5S hybrid ribosomes are marked with red dots and those showing varying accessibility are indicated with yellow dots. Numbers on the right side of the gels indicate the position of the respective nucleotides within wt 5S rRNA (panel **b**) or 23S rRNA (panel **c**) primary structures. The uncropped gels can be found in the Source data file. Representative gels of two independent experiments are shown in panels **b** and **c**.

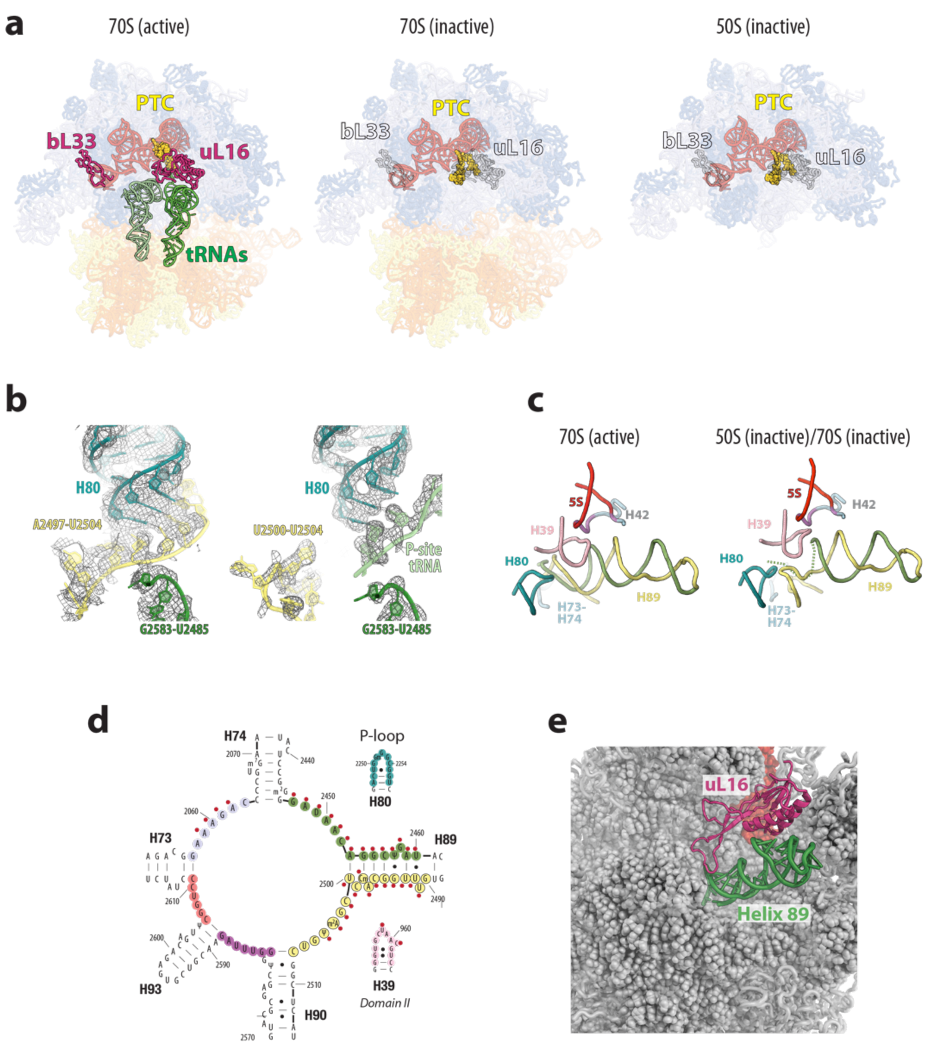

Supplementary Fig. 5 | PTC structure is dramatically distorted in the aberrant 50S subunits and inactive 70S ribosomes containing hybrid 23S-cp5S rRNA. a Cryo-EM structures of the active 70S ribosomes carrying P- and A-site tRNAs, 70S inactive ribosomes that lack tRNAs, and the unassociated 50S subunits. tRNAs are in green, r-proteins bL33 and uL16 are in purple. The proteins lacking in inactive ribosomes and aberrant 50S subunits are shown as gray contours. **b** Cryo-EM density (mesh) showing the altered path of 23S rRNA in the PTC of the vacant ribosome (left panel), which is similar to the one in the defective 50S subunits, relative to that in the functional ribosome bound with P-tRNA (light green; right panel). The colors of the RNA backbone match those in the diagram shown in panel d. Vacant and tRNA-bound 70S cryo-EM maps were sharpened by applying B-factors of -40 Å^2 and -50 Å^2, respectively. Both maps are shown at σ = 4.5. **c** Comparison of the rRNA structure in the PTC of the active ribosomes (top) with the perturbed rRNA structure in the PTC of the aberrant 50S subunits and inactive ribosomes. In the active ribosome, the first helical turn of H89 is base-paired, whereas this segment is unwound in the aberrant 50S subunits. Low cryo-EM density of the segment (2450-2455; pale green dotted line) suggests structural disorder of the segment and prevents accurate modeling. The colors of the rRNA backbone match those shown in the secondary structure diagram in panel **d**. **d** Secondary structure diagram of the PTC rRNA segments. **e** Distortion of H89 could destabilize binding of uL16 due to their proximity in the ribosome.

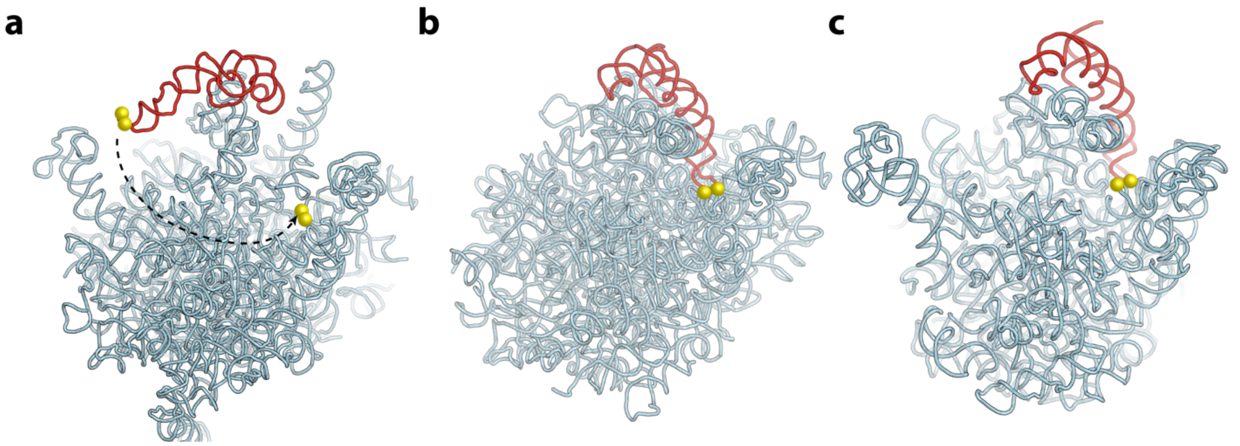

Supplementary Fig. 6 | The initial binding mode of 5S rRNA to the large subunit assembly intermediates that requires subsequent 5S rRNA rotation is impossible in the 23S-cp5S ribosomes. The difference in position of 5S rRNA (red) observed in (**a**) immature (PDB ID 4V7F)[7] and (**b**) fully assembled (PDB ID 4V88)[8] eukaryotic large ribosomal subunits or (**c**) in the fully-assembled bacterial 50S ribosomal subunit (PDB ID 4YBB)[9]. 23S rRNA is light blue; r-proteins have been removed for clarity. The 5S rRNA and 23S rRNA nucleotides connected with the short tethers in the 23S-cp5S rRNA ribosome are marked by yellow spheres.

SUPLLEMENTARY REFERENCES

1 Quan, S., Skovgaard, O., McLaughlin, R. E., Buurman, E. T. & Squires, C. L. Markerless *Escherichia coli rrn* deletion strains for genetic determination of ribosomal binding sites. *G3)* **5**, 2555-2557 (2015).

2 Orelle, C. *et al.* Protein synthesis by ribosomes with tethered subunits. *Nature* **524**, 119-124 (2015).

3 Yanisch-Perron, C., Vieira, J. & Messing, J. Improved M13 phage cloning vectors and host strains: nucleotide sequences of the M13mp18 and pUC19 vectors. *Gene* **33**, 103-119 (1985).

4 Kusters, J. G., Jager, E. J. & van der Zeijst, B. A. Improvement of the cloning linker of the bacterial expression vector pEX. *Nucleic Acids Res.* **17**, 8007 (1989).

5 Datsenko, K. A. & Wanner, B. L. One-step inactivation of chromosomal genes in Escherichia coli K-12 using PCR products. *Proc. Natl. Acad. Sci. USA* **97**, 6640-6645 (2000).

6 Zaporojets, D., French, S. & Squires, C. L. Products transcribed from rearranged rrn genes of Escherichia coli can assemble to form functional ribosomes. *J. Bacteriol.* **185**, 6921-6927 (2003).

7 Leidig, C. *et al.* 60S ribosome biogenesis requires rotation of the 5S ribonucleoprotein particle. *Nat. Commun.* **5**, 3491 (2014).

8 Ben-Shem, A. *et al.* The structure of the eukaryotic ribosome at 3.0 A resolution. *Science* **334**, 1524-1529 (2011)

9 Noeske, J. *et al.* High-resolution structure of the Escherichia coli ribosome. *Nat. Struct. Molec. Biol.* **22**, 336-341 (2015).
