# Supplementary material for: Ribosome engineering reveals the importance of 5S rRNA autonomy for ribosome assembly
Source: Nat Commun. 2020 Jun 9;11:2900. doi: 10.1038/s41467-020-16694-8 (PMC7283268; doi:10.1038/s41467-020-16694-8)
Supplement: Supplementary file 4 — Source Data [file 41467_2020_16694_MOESM4_ESM.zip › New Folder With Items/248051_2_data_set_4606476_q60yb4.pdf]

pAM552

```
LOCUS       Exported                7452 bp ds-DNA     circular SYN 30-AUG-2019
DEFINITION  synthetic circular DNA
ACCESSION   .
VERSION     .
KEYWORDS    .
SOURCE      synthetic DNA construct
  ORGANISM  synthetic DNA construct
REFERENCE   1  (bases 1 to 7452)
  AUTHORS   Alexander S. Mankin
  TITLE     Direct Submission
  JOURNAL   Exported Friday, Aug 30, 2019 from SnapGene 4.3.10
            https://www.snapgene.com
FEATURES             Location/Qualifiers
     source          1..7452
                     /organism="synthetic DNA construct"
                     /mol_type="other DNA"
     promoter        9..148
                     /locus_tag="pL promoter"
                     /label=pL promoter
     -35_signal      107..112
                     /locus_tag="-35"
                     /label=-35
     -10_signal      130..135
                     /locus_tag="-10"
                     /label=-10
     rRNA            320..1861
                     /locus_tag="16S rRNA"
                     /label=16S rRNA
     tRNA            2033..2108
                     /locus_tag="Glu tRNA"
                     /label=Glu tRNA
     rRNA            2302..5205
                     /locus_tag="23S rRNA"
                     /label=23S rRNA
     rRNA            5299..5417
                     /locus_tag="5S rRNA"
                     /label=5S rRNA
     terminator      5418..5504
                     /gene="Escherichia coli rrnB"
                     /label=rrnB T1 terminator
                     /note="transcription terminator T1 from the E. coli rrnB
                     gene"
     terminator      5596..5623
                     /label=rrnB T2 terminator
                     /note="transcription terminator T2 from the E. coli rrnB
                     gene"
     promoter        5684..5788
                     /gene="bla"
                     /label=AmpR promoter
     CDS             5789..6649
                     /codon_start=1
                     /gene="bla"
                     /product="beta-lactamase"
```

```
                     /label=AmpR
                     /note="confers resistance to ampicillin, carbenicillin, and
                     related antibiotics"
                     /translation="MSIQHFRVALIPFFAAFCLPVFAHPETLVKVKDAEDQLGARVGYI
                     ELDLNSGKILESFRPEERFPMMSTFKVLLCGAVLSRVDAGQEQLGRRIHYSQNDLVEYS
                     PVTEKHLTDGMTVRELCSAAITMSDNTAANLLLTTIGGPKELTAFLHNMGDHVTRLDRW
                     EPELNEAIPNDERDTTMPAAMATTLRKLLTGELLTLASRQQLIDWMEADKVAGPLLRSA
                     LPASWFIADKSGAGERGSRGIIAALGPDGKPSRIVVIYTTGSQATMDERNRQIAEIGAS
                     LIKHW"
     rep_origin      6819..7407
                     /direction=RIGHT
                     /label=ori
                     /note="high-copy-number ColE1/pMB1/pBR322/pUC origin of
                     replication"
ORIGIN
        1 GCGGCCGCGA TCTCTCACCT ACCAAACAAT GCCCCCCTGC AAAAAATAAA TTCATATAAA
       61 AAACATACAG ATAACCATCT GCGGTGATAA ATTATCTCTG GCGGTGTTGA CATAAATACC
      121 ACTGGCGGTG ATACTGAGCA CGGGTACCGG CCGCTGAGAA AAAGCGAAGC GGCACTGCTC
      181 TTTAACAATT TATCAGACAA TCTGTGTGGG CACTCGAAGA TACGGATTCT TAACGTCGCA
      241 AGACGAAAAA TGAATACCAA GTCTCAAGAG TGAACACGTA ATTCATTACG AAGTTTAATT
      301 CTTTGAGCGT CAAACTTTTA AATTGAAGAG TTTGATCATG GCTCAGATTG AACGCTGGCG
      361 GCAGGCCTAA CACATGCAAG TCGAACGGTA ACAGGAAGAA GCTTGCTTCT TTGCTGACGA
      421 GTGGCGGACG GGTGAGTAAT GTCTGGGAAA CTGCCTGATG GAGGGGGATA ACTACTGGAA
      481 ACGGTAGCTA ATACCGCATA ACGTCGCAAG ACCAAAGAGG GGGACCTTCG GGCCTCTTGC
      541 CATCGGATGT GCCCAGATGG GATTAGCTAG TAGGTGGGGT AACGGCTCAC CTAGGCGACG
      601 ATCCCTAGCT GGTCTGAGAG GATGACCAGC CACACTGGAA CTGAGACACG GTCCAGACTC
      661 CTACGGGAGG CAGCAGTGGG GAATATTGCA CAATGGGCGC AAGCCTGATG CAGCCATGCC
      721 GCGTGTATGA AGAAGGCCTT CGGGTTGTAA AGTACTTTCA GCGGGGAGGA AGGGAGTAAA
      781 GTTAATACCT TTGCTCATTG ACGTTACCCG CAGAAGAAGC ACCGGCTAAC TCCGTGCCAG
      841 CAGCCGCGGT AATACGGAGG GTGCAAGCGT TAATCGGAAT TACTGGGCGT AAAGCGCACG
      901 CAGGCGGTTT GTTAAGTCAG ATGTGAAATC CCCGGGCTCA ACCTGGGAAC TGCATCTGAT
      961 ACTGGCAAGC TTGAGTCTCG TAGAGGGGGG TAGAATTCCA GGTGTAGCGG TGAAATGCGT
     1021 AGAGATCTGG AGGAATACCG GTGGCGAAGG CGGCCCCCTG GACGAAGACT GACGCTCAGG
     1081 TGCGAAAGCG TGGGGAGCAA ACAGGATTAG ATACCCTGGT AGTCCACGCC GTAAACGATG
     1141 TCGACTTGGA GGTTGTGCCC TTGAGGCGTG GCTTCCGGAG CTAACGCGTT AAGTCGACCG
     1201 CCTGGGGAGT ACGGCCGCAA GGTTAAAACT CAAATGAATT GACGGGGGCC CGCACAAGCG
     1261 GTGGAGCATG TGGTTTAATT CGATGCAACG CGAAGAACCT TACCTGGTCT TGACATCCAC
     1321 GGAAGTTTTC AGAGATGAGA ATGTGCCTTC GGGAACCGTG AGACAGGTGC TGCATGGCTG
     1381 TCGTCAGCTC GTGTTGTGAA ATGTTGGGTT AAGTCCCGCA ACGAGCGCAA CCCTTATCCT
     1441 TTGTTGCCAG CGGTCCGGCC GGGAACTCAA AGGAGACTGC CAGTGATAAA CTGGAGGAAG
     1501 GTGGGGATGA CGTCAAGTCA TCATGGCCCT TACGACCAGG GCTACACACG TGCTACAATG
     1561 GCGCATACAA AGAGAAGCGA CCTCGCGAGA GCAAGCGGAC CTCATAAAGT GCGTCGTAGT
     1621 CCGGATTGGA GTCTGCAACT CGACTCCATG AAGTCGGAAT CGCTAGTAAT CGTGGATCAG
     1681 AATGCCACGG TGAATACGTT CCCGGGCCTT GTACACACCG CCCGTCACAC CATGGGAGTG
     1741 GGTTGCAAAA GAAGTAGGTA GCTTAACCTT CGGGAGGGCG CTTACCACTT TGTGATTCAT
     1801 GACTGGGGTG AAGTCGTAAC AAGGTAACCG TAGGGGAACC TGCGGTTGGA TCACCTCCTT
     1861 ACCTTAAAGA AGCGTACTTT GTAGTGCTCA CACAGATTGT CTGATAGAAA GTGAAAAGCA
     1921 AGGCGTTTAC GCGTTGGGAG TGAGGCTGAA GAGAATAAGG CCGTTCGCTT TCTATTAATG
     1981 AAAGCTCACC CTACACGAAA ATATCACGCA ACGCGTGATA AGCAATTTTC GTGTCCCCTT
     2041 CGTCTAGAGG CCCAGGACAC CGCCCTTTCA CGGCGGTAAC AGGGGTTCGA ATCCCCTAGG
     2101 GGACGCCACT TGCTGGTTTG TGAGTGAAAG TCGCCGACCT TAATATCTCA AAACTCATCT
     2161 TCGGGTGATG TTTGAGATAT TTGCTCTTTA AAAATCTGGA TCAAGCTGAA AATTGAAACA
     2221 CTGAACAACG AGAGTTGTTC GTGAGTCTCT CAAATTTTCG CAACACGATG ATGAATCGAA
     2281 AGAAACATCT TCGGGTTGTG AGGTTAAGCG ACTAAGCGTA CACGGTGGAT GCCCTGGCAG
     2341 TCAGAGGCGA TGAAGGACGT GCTAATCTGC GATAAGCGTC GGTAAGGTGA TATGAACCGT
     2401 TATAACCGGC GATTTCCGAA TGGGGAAACC CAGTGTGTTT CGACACACTA TCATTAACTG
```

2461 AATCCATAGG TTAATGAGGC GAACCGGGGG AACTGAAACA TCTAAGTACC CCGAGGAAAA
2521 GAAATCAACC GAGATTCCCC CAGTAGCGGC GAGCGAACGG GGAGCAGCCC AGAGCCTGAA
2581 TCAGTGTGTG TGTTAGTGGA AGCGTCTGGA AAGGCGCGCG ATACAGGGTG ACAGCCCCGT
2641 ACACAAAAAT GCACATGCTG TGAGCTCGAT GAGTAGGGCG GGACACGTGG TATCCTGTCT
2701 GAATATGGGG GGACCATCCT CCAAGGCTAA ATACTCCTGA CTGACCGATA GTGAACCAGT
2761 ACCGTGAGGG AAAGGCGAAA AGAACCCCGG CGAGGGGAGT GAAAAAGAAC CTGAAACCGT
2821 GTACGTACAA GCAGTGGGAG CACGCTTAGG CGTGTGACTG CGTACCTTTT GTATAATGGG
2881 TCAGCGACTT ATATTCTGTA GCAAGGTTAA CCGAATAGGG GAGCCGAAGG GAAACCGAGT
2941 CTTAACTGGG CGTTAAGTTG CAGGGTATAG ACCCGAAACC CGGTGATCTA GCCATGGGCA
3001 GGTTGAAGGT TGGGTAACAC TAACTGGAGG ACCGAACCGA CTAATGTTGA AAAATTAGCG
3061 GATGACTTGT GGCTGGGGGT GAAAGGCCAA TCAAACCGGG AGATAGCTGG TTCTCCCCGA
3121 AAGCTATTTA GGTAGCGCCT CGTGAATTCA TCTCCGGGGG TAGAGCACTG TTTCGGCAAG
3181 GGGGTCATCC CGACTTACCA ACCCGATGCA AACTGCGAAT ACCGGAGAAT GTTATCACGG
3241 GAGACACACG GCGGGTGCTA ACGTCCGTCG TGAAGAGGGA AACAACCCAG ACCGCCAGCT
3301 AAGGTCCCAA AGTCATGGTT AAGTGGGAAA CGATGTGGGA AGGCCCAGAC AGCCAGGATG
3361 TTGGCTTAGA AGCAGCCATC ATTTAAAGAA AGCGTAATAG CTCACTGGTC GAGTCGGCCT
3421 GCGCGGAAGA TGTAACGGGG CTAAACCATG CACCGAAGCT GCGGCAGCGA CGCTTATGCG
3481 TTGTTGGGTA GGGGAGCGTT CTGTAAGCCT GCGAAGGTGT GCTGTGAGGC ATGCTGGAGG
3541 TATCAGAAGT GCGAATGCTG ACATAAGTAA CGATAAAGCG GGTGAAAAGC CCGCTCGCCG
3601 GAAGACCAAG GGTTCCTGTC CAACGTTAAT CGGGGCAGGG TGAGTCGACC CCTAAGGCGA
3661 GGCCGAAAGG CGTAGTCGAT GGGAAACAGG TTAATATTCC TGTACTTGGT GTTACTGCGA
3721 AGGGGGGACG GAGAAGGCTA TGTTGGCCGG GCGACGGTTG TCCCGGTTTA AGCGTGTAGG
3781 CTGGTTTTCC AGGCAAATCC GGAAAATCAA GGCTGAGGCG TGATGACGAG GCACTACGGT
3841 GCTGAAGCAA CAAATGCCCT GCTTCCAGGA AAAGCCTCTA AGCATCAGGT AACATCAAAT
3901 CGTACCCCAA ACCGACACAG GTGGTCAGGT AGAGAATACC AAGGCGCTTG AGAGAACTCG
3961 GGTGAAGGAA CTAGGCAAAA TGGTGCCGTA ACTTCGGGAG AAGGCACGCT GATATGTAGG
4021 TGAGGTCCCT CGCGGATGGA GCTGAAATCA GTCGAAGATA CCAGCTGGCT GCAACTGTTT
4081 ATTAAAAACA CAGCACTGTG CAAACACGAA AGTGGACGTA TACGGTGTGA CGCCTGCCCG
4141 GTGCCGGAAG GTTAATTGAT GGGGTTAGCG CAAGCGAAGC TCTTGATCGA AGCCCCGGTA
4201 AACGGCGGCC GTAACTATAA CGGTCCTAAG GTAGCGAAAT TCCTTGTCGG GTAAGTTCCG
4261 ACCTGCACGA ATGGCGTAAT GATGGCCAGG CTGTCTCCAC CCGAGACTCA GTGAAATTGA
4321 ACTCGCTGTG AAGATGCAGT GTACCCGCGG CAAGACGGAA AGACCCCGTG AACCTTTACT
4381 ATAGCTTGAC ACTGAACATT GAGCCTTGAT GTGTAGGATA GGTGGGAGGC TTTGAAGTGT
4441 GGACGCCAGT CTGCATGGAG CCGACCTTGA AATACCACCC TTTAATGTTT GATGTTCTAA
4501 CGTTGACCCG TAATCCGGGT TGCGGACAGT GTCTGGTGGG TAGTTTGACT GGGGCGGTCT
4561 CCTCCTAAAG AGTAACGGAG GAGCACGAAG GTTGGCTAAT CCTGGTCGGA CATCAGGAGG
4621 TTAGTGCAAT GGCATAAGCC AGCTTGACTG CGAGCGTGAC GGCGCGAGCA GGTGCGAAAG
4681 CAGGTCATAG TGATCCGGTG GTTCTGAATG GAAGGGCCAT CGCTCAACGG ATAAAAGGTA
4741 CTCCGGGGAT AACAGGCTGA TACCGCCCAA GAGTTCATAT CGACGGCGGT GTTTGGCACC
4801 TCGATGTCGG CTCATCACAT CCTGGGGCTG AAGTAGGTCC CAAGGGTATG GCTGTTCGCC
4861 ATTTAAAGTG GTACGCGAGC TGGGTTTAGA ACGTCGTGAG ACAGTTCGGT CCCTATCTGC
4921 CGTGGGCGCT GGAGAACTGA GGGGGGCTGC TCCTAGTACG AGAGGACCGG AGTGGACGCA
4981 TCACTGGTGT TCGGGTTGTC ATGCCAATGG CACTGCCCGG TAGCTAAATG CGGAAGAGAT
5041 AAGTGCTGAA AGCATCTAAG CACGAAACTT GCCCCGAGAT GAGTTCTCCC TGACCCTTTA
5101 AGGGTCCTGA AGGAACGTTG AAGACGACGA CGTTGATAGG CCGGGTGTGT AAGCGCAGCG
5161 ATGCGTTGAG CTAACCGGTA CTAATGAACC GTGAGGCTTA ACCTTACAAC GCCGAAGCTG
5221 TTTTGGCGGA TGAGAGAAGA TTTTCAGCCT GATACAGATT AAATCAGAAC GCAGAAGCGG
5281 TCTGATAAAA CAGAATTTGC CTGGCGGCAG TAGCGCGGTG GTCCCACCTG ACCCCATGCC
5341 GAACTCAGAA GTGAAACGCC GTAGCGCCGA TGGTAGTGTG GGGTCTCCCC ATGCGAGAGT
5401 AGGGAACTGC CAGGCATCAA ATAAAACGAA AGGCTCAGTC GAAAGACTGG GCCTTTCGTT
5461 TTATCTGTTG TTTGTCGGTG AACGCTCTCC TGAGTAGGAC AAATCCGCCG GGAGCGGATT
5521 TGAACGTTGC GAAGCAACGG CCCGGAGGGT GGCGGGCAGG ACGCCCGCCA TAAACTGCCA
5581 GGCATCAAAT TAAGCAGAAG GCCATCCTGA CGGATGGCCT TTTTGCGTTT CTACAAACTC
5641 TTCCTGTCGT CATATCTACA AGCCGGCGCG CCCGGGAAAT GTGCGCGGAA CCCCTATTTG
5701 TTTATTTTTC TAAATACATT CAAATATGTA TCCGCTCATG AGACAATAAC CCTGATAAAT
5761 GCTTCAATAA TATTGAAAAA GGAAGAGTAT GAGTATTCAA CATTTCCGTG TCGCCCTTAT
5821 TCCCTTTTTT GCGGCATTTT GCCTTCCTGT TTTTGCTCAC CCAGAAACGC TGGTGAAAGT

```
     5881 AAAAGATGCT GAAGATCAGT TGGGTGCACG AGTGGGTTAC ATCGAACTGG ATCTCAACAG
     5941 CGGTAAGATC CTTGAGAGTT TTCGCCCCGA AGAACGTTTT CCAATGATGA GCACTTTTAA
     6001 AGTTCTGCTA TGTGGCGCGG TATTATCCCG TGTTGACGCC GGGCAAGAGC AACTCGGTCG
     6061 CCGCATACAC TATTCTCAGA ATGACTTGGT TGAGTACTCA CCAGTCACAG AAAAGCATCT
     6121 TACGGATGGC ATGACAGTAA GAGAATTATG CAGTGCTGCA ATAACCATGA GTGATAACAC
     6181 TGCGGCCAAC TTACTTCTGA CAACGATCGG AGGACCGAAG GAGCTAACCG CTTTTTTGCA
     6241 CAACATGGGG GATCATGTAA CTCGCCTTGA TCGTTGGGAA CCGGAGCTGA ATGAAGCCAT
     6301 ACCAAACGAC GAGCGTGACA CCACGATGCC TGCAGCAATG GCAACAACGT TGCGCAAACT
     6361 ATTAACTGGC GAACTACTTA CTCTAGCTTC CCGGCAACAA TTAATAGACT GGATGGAGGC
     6421 GGATAAAGTT GCAGGACCAC TTCTGCGCTC GGCCCTTCCG GCTAGCTGGT TTATTGCTGA
     6481 TAAATCTGGA GCCGGTGAGC GTGGGTCTCG CGGTATCATT GCAGCACTGG GGCCAGATGG
     6541 TAAGCCCTCC CGTATCGTAG TTATCTACAC GACGGGGAGT CAGGCAACTA TGGATGAACG
     6601 AAATAGACAG ATCGCTGAGA TAGGTGCCTC ACTGATTAAG CATTGGTAAC TGCAGACCAA
     6661 GTTTACTCAT ATATACTTTA GATTGATTTA AAACTTCATT TTTAATTTAA AAGGATCTAG
     6721 GTGAAGATCC TTTTTGATAA TCTCATGACC AAAATCCCTT AACGTGAGTT TTCGTTCCAC
     6781 TGAGCGTCAG ACCCCGTAGA AAAGATCAAA GGATCTTCTT GAGATCCTTT TTTTCTGCGC
     6841 GTAATCTGCT GCTTGCAAAC AAAAAAACCA CCGCTACCAG CGGTGGTTTG TTTGCCGGAT
     6901 CAAGAGCTAC CAACTCTTTT TCCGAAGGTA ACTGGCTTCA GCAGAGCGCA GATACCAAAT
     6961 ACTGTCCTTC TAGTGTAGCC GTAGTTAGGC CACCACTTCA AGAACTCTGT AGCACCGCCT
     7021 ACATACCTCG CTCTGCTAAT CCTGTTACCA GTGGCTGCTG CCAGTGGCGA TAAGTCGTGT
     7081 CTTACCGGGT TGGACTCAAG ACGATAGTTA CCGGATAAGG CGCAGCGGTC GGGCTGAACG
     7141 GGGGGTTCGT GCACACAGCC CAGCTTGGAG CGAACGACCT ACACCGAACT GAGATACCTA
     7201 CAGCGTGAGC TATGAGAAAG CGCCACGCTT CCCGAAGGGA GAAAGGCGGA CAGGTATCCG
     7261 GTAAGCGGCA GGGTCGGAAC AGGAGAGCGC ACGAGGGAGC TTCCAGGGGG AAACGCCTGG
     7321 TATCTTTATA GTCCTGTCGG GTTTCGCCAC CTCTGACTTG AGCGTCGATT TTTGTGATGC
     7381 TCGTCAGGGG GGCGGAGCCT ATGGAAAAAC GCCAGCAACG CGGCCTTTTT ACGGTTCCTG
     7441 GCCTTTTGCT GG
//
```

pDH42 (CUG/A)

```
LOCUS       Exported              7461 bp ds-DNA     circular SYN 30-AUG-2019
DEFINITION  synthetic circular DNA
ACCESSION   .
VERSION     .
KEYWORDS    .
SOURCE      synthetic DNA construct
  ORGANISM  synthetic DNA construct
REFERENCE   1  (bases 1 to 7461)
  AUTHORS   Alexander S. Mankin
  TITLE     Direct Submission
  JOURNAL   Exported Friday, Aug 30, 2019 from SnapGene 4.3.10
            https://www.snapgene.com
FEATURES             Location/Qualifiers
     source          1..7461
                     /organism="synthetic DNA construct"
                     /lab_host="Alexander S. Mankin"
                     /mol_type="other DNA"
     promoter        9..148
                     /locus_tag="pL promoter"
                     /label=pL promoter
     -35_signal      107..112
                     /locus_tag="-35"
                     /label=-35
```

```
-10_signal      130..135
                /locus_tag="-10"
                /label=-10
rRNA            320..1861
                /locus_tag="16S rRNA"
                /label=16S rRNA
tRNA            2033..2108
                /locus_tag="Glu tRNA"
                /label=Glu tRNA
rRNA            2302..3327
                /locus_tag="23S rRNA first part"
                /label=23S rRNA first part
rRNA            3328..3330
                /locus_tag="Linker 1"
                /label=Linker 1
rRNA            3331..3453
                /locus_tag="cp5S rRNA"
                /label=cp5S rRNA
rRNA            3362..3367
                /locus_tag="cp linker"
                /label=cp linker
rRNA            3454
                /locus_tag="Linker 2"
                /label=Linker 2
rRNA            3455..5332
                /locus_tag="23S rRNA second part"
                /label=23S rRNA second part
terminator      5428..5514
                /gene="Escherichia coli rrnB"
                /label=rrnB T1 terminator
                /note="transcription terminator T1 from the E. coli rrnB
                gene"
terminator      5606..5633
                /label=rrnB T2 terminator
                /note="transcription terminator T2 from the E. coli rrnB
                gene"
promoter        5693..5797
                /gene="bla"
                /label=AmpR promoter
CDS             5798..6658
                /codon_start=1
                /gene="bla"
                /product="beta-lactamase"
                /label=AmpR
                /note="confers resistance to ampicillin, carbenicillin, and
                related antibiotics"
                /translation="MSIQHFRVALIPFFAAFCLPVFAHPETLVKVKDAEDQLGARVGYI
                ELDLNSGKILESFRPEERFPMMSTFKVLLCGAVLSRVDAGQEQLGRRIHYSQNDLVEYS
                PVTEKHLTDGMTVRELCSAAITMSDNTAANLLLTTIGGPKELTAFLHNMGDHVTRLDRW
                EPELNEAIPNDERDTTMPAAMATTLRKLLTGELLTLASRQQLIDWMEADKVAGPLLRSA
                LPASWFIADKSGAGERGSRGIIAALGPDGKPSRIVVIYTTGSQATMDERNRQIAEIGAS
                LIKHW"
rep_origin      6828..7416
                /direction=RIGHT
                /label=ori
                /note="high-copy-number ColE1/pMB1/pBR322/pUC origin of
```

replication"

ORIGIN

```
        1 GCGGCCGCGA TCTCTCACCT ACCAAACAAT GCCCCCCTGC AAAAAATAAA TTCATATAAA
       61 AAACATACAG ATAACCATCT GCGGTGATAA ATTATCTCTG GCGGTGTTGA CATAAATACC
      121 ACTGGCGGTG ATACTGAGCA CGGGTACCGG CCGCTGAGAA AAAGCGAAGC GGCACTGCTC
      181 TTTAACAATT TATCAGACAA TCTGTGTGGG CACTCGAAGA TACGGATTCT TAACGTCGCA
      241 AGACGAAAAA TGAATACCAA GTCTCAAGAG TGAACACGTA ATTCATTACG AAGTTTAATT
      301 CTTTGAGCGT CAAACTTTTA AATTGAAGAG TTTGATCATG GCTCAGATTG AACGCTGGCG
      361 GCAGGCCTAA CACATGCAAG TCGAACGGTA ACAGGAAGAA GCTTGCTTCT TTGCTGACGA
      421 GTGGCGGACG GGTGAGTAAT GTCTGGGAAA CTGCCTGATG GAGGGGGATA ACTACTGGAA
      481 ACGGTAGCTA ATACCGCATA ACGTCGCAAG ACCAAAGAGG GGGACCTTCG GGCCTCTTGC
      541 CATCGGATGT GCCCAGATGG GATTAGCTAG TAGGTGGGGT AACGGCTCAC CTAGGCGACG
      601 ATCCCTAGCT GGTCTGAGAG GATGACCAGC CACACTGGAA CTGAGACACG GTCCAGACTC
      661 CTACGGGAGG CAGCAGTGGG GAATATTGCA CAATGGGCGC AAGCCTGATG CAGCCATGCC
      721 GCGTGTATGA AGAAGGCCTT CGGGTTGTAA AGTACTTTCA GCGGGGAGGA AGGGAGTAAA
      781 GTTAATACCT TTGCTCATTG ACGTTACCCG CAGAAGAAGC ACCGGCTAAC TCCGTGCCAG
      841 CAGCCGCGGT AATACGGAGG GTGCAAGCGT TAATCGGAAT TACTGGGCGT AAAGCGCACG
      901 CAGGCGGTTT GTTAAGTCAG ATGTGAAATC CCCGGGCTCA ACCTGGGAAC TGCATCTGAT
      961 ACTGGCAAGC TTGAGTCTCG TAGAGGGGGG TAGAATTCCA GGTGTAGCGG TGAAATGCGT
     1021 AGAGATCTGG AGGAATACCG GTGGCGAAGG CGGCCCCCTG GACGAAGACT GACGCTCAGG
     1081 TGCGAAAGCG TGGGGAGCAA ACAGGATTAG ATACCCTGGT AGTCCACGCC GTAAACGATG
     1141 TCGACTTGGA GGTTGTGCCC TTGAGGCGTG GCTTCCGGAG CTAACGCGTT AAGTCGACCG
     1201 CCTGGGGAGT ACGGCCGCAA GGTTAAAACT CAAATGAATT GACGGGGGCC CGCACAAGCG
     1261 GTGGAGCATG TGGTTTAATT CGATGCAACG CGAAGAACCT TACCTGGTCT TGACATCCAC
     1321 GGAAGTTTTC AGAGATGAGA ATGTGCCTTC GGGAACCGTG AGACAGGTGC TGCATGGCTG
     1381 TCGTCAGCTC GTGTTGTGAA ATGTTGGGTT AAGTCCCGCA ACGAGCGCAA CCCTTATCCT
     1441 TTGTTGCCAG CGGTCCGGCC GGGAACTCAA AGGAGACTGC CAGTGATAAA CTGGAGGAAG
     1501 GTGGGGATGA CGTCAAGTCA TCATGGCCCT TACGACCAGG GCTACACACG TGCTACAATG
     1561 GCGCATACAA AGAGAAGCGA CCTCGCGAGA GCAAGCGGAC CTCATAAAGT GCGTCGTAGT
     1621 CCGGATTGGA GTCTGCAACT CGACTCCATG AAGTCGGAAT CGCTAGTAAT CGTGGATCAG
     1681 AATGCCACGG TGAATACGTT CCCGGGCCTT GTACACACCG CCCGTCACAC CATGGGAGTG
     1741 GGTTGCAAAA GAAGTAGGTA GCTTAACCTT CGGGAGGGCG CTTACCACTT TGTGATTCAT
     1801 GACTGGGGTG AAGTCGTAAC AAGGTAACCG TAGGGGAACC TGCGGTTGGA TCACCTCCTT
     1861 ACCTTAAAGA AGCGTACTTT GTAGTGCTCA CACAGATTGT CTGATAGAAA GTGAAAAGCA
     1921 AGGCGTTTAC GCGTTGGGAG TGAGGCTGAA GAGAATAAGG CCGTTCGCTT TCTATTAATG
     1981 AAAGCTCACC CTACACGAAA ATATCACGCA ACGCGTGATA AGCAATTTTC GTGTCCCCTT
     2041 CGTCTAGAGG CCCAGGACAC CGCCCTTTCA CGGCGGTAAC AGGGGTTCGA ATCCCCTAGG
     2101 GGACGCCACT TGCTGGTTTG TGAGTGAAAG TCGCCGACCT TAATATCTCA AAACTCATCT
     2161 TCGGGTGATG TTTGAGATAT TTGCTCTTTA AAAATCTGGA TCAAGCTGAA AATTGAAACA
     2221 CTGAACAACG AGAGTTGTTC GTGAGTCTCT CAAATTTTCG CAACACGATG ATGAATCGAA
     2281 AGAAACATCT TCGGGTTGTG AGGTTAAGCG ACTAAGCGTA CACGGTGGAT GCCCTGGCAG
     2341 TCAGAGGCGA TGAAGGACGT GCTAATCTGC GATAAGCGTC GGTAAGGTGA TATGAACCGT
     2401 TATAACCGGC GATTTCCGAA TGGGGAAACC CAGTGTGTTT CGACACACTA TCATTAACTG
     2461 AATCCATAGG TTAATGAGGC GAACCGGGGG AACTGAAACA TCTAAGTACC CCGAGGAAAA
     2521 GAAATCAACC GAGATTCCCC CAGTAGCGGC GAGCGAACGG GGAGCAGCCC AGAGCCTGAA
     2581 TCAGTGTGTG TGTTAGTGGA AGCGTCTGGA AAGGCGCGCG ATACAGGGTG ACAGCCCCGT
     2641 ACACAAAAAT GCACATGCTG TGAGCTCGAT GAGTAGGGCG GGACACGTGG TATCCTGTCT
     2701 GAATATGGGG GGACCATCCT CCAAGGCTAA ATACTCCTGA CTGACCGATA GTGAACCAGT
     2761 ACCGTGAGGG AAAGGCGAAA AGAACCCCGG CGAGGGGAGT GAAAAAGAAC CTGAAACCGT
     2821 GTACGTACAA GCAGTGGGAG CACGCTTAGG CGTGTGACTG CGTACCTTTT GTATAATGGG
     2881 TCAGCGACTT ATATTCTGTA GCAAGGTTAA CCGAATAGGG GAGCCGAAGG GAAACCGAGT
     2941 CTTAACTGGG CGTTAAGTTG CAGGGTATAG ACCCGAAACC CGGTGATCTA GCCATGGGCA
     3001 GGTTGAAGGT TGGGTAACAC TAACTGGAGG ACCGAACCGA CTAATGTTGA AAAATTAGCG
     3061 GATGACTTGT GGCTGGGGGT GAAAGGCCAA TCAAACCGGG AGATAGCTGG TTCTCCCCGA
     3121 AAGCTATTTA GGTAGCGCCT CGTGAATTCA TCTCCGGGGG TAGAGCACTG TTTCGGCAAG
     3181 GGGGTCATCC CGACTTACCA ACCCGATGCA AACTGCGAAT ACCGGAGAAT GTTATCACGG
     3241 GAGACACACG GCGGGTGCTA ACGTCCGTCG TGAAGAGGGA AACAACCCAG ACCGCCAGCT
```

3301 AAGGTCCCAA AGTCATGGTT AAGTGGGCTG CTCCCCATGC GAGAGTAGGG AACTGCCAGG
3361 CCTTCGGGCC TGGCGGCAGT AGCGCGGTGG TCCCACCTGA CCCCATGCCG AACTCAGAAG
3421 TGAAACGCCG TAGCGCCGAT GGTAGTGTGG GGTAAAACGA TGTGGGAAGG CCCAGACAGC
3481 CAGGATGTTG GCTTAGAAGC AGCCATCATT TAAAGAAAGC GTAATAGCTC ACTGGTCGAG
3541 TCGGCCTGCG CGGAAGATGT AACGGGGCTA AACCATGCAC CGAAGCTGCG GCAGCGACGC
3601 TTATGCGTTG TTGGGTAGGG GAGCGTTCTG TAAGCCTGCG AAGGTGTGCT GTGAGGCATG
3661 CTGGAGGTAT CAGAAGTGCG AATGCTGACA TAAGTAACGA TAAAGCGGGT GAAAAGCCCG
3721 CTCGCCGGAA GACCAAGGGT TCCTGTCCAA CGTTAATCGG GGCAGGGTGA GTCGACCCCT
3781 AAGGCGAGGC CGAAAGGCGT AGTCGATGGG AAACAGGTTA ATATTCCTGT ACTTGGTGTT
3841 ACTGCGAAGG GGGGACGGAG AAGGCTATGT TGGCCGGGCG ACGGTTGTCC CGGTTTAAGC
3901 GTGTAGGCTG GTTTTCCAGG CAAATCCGGA AAATCAAGGC TGAGGCGTGA TGACGAGGCA
3961 CTACGGTGCT GAAGCAACAA ATGCCCTGCT TCCAGGAAAA GCCTCTAAGC ATCAGGTAAC
4021 ATCAAATCGT ACCCCAAACC GACACAGGTG GTCAGGTAGA GAATACCAAG GCGCTTGAGA
4081 GAACTCGGGT GAAGGAACTA GGCAAAATGG TGCCGTAACT TCGGGAGAAG GCACGCTGAT
4141 ATGTAGGTGA GGTCCCTCGC GGATGGAGCT GAAATCAGTC GAAGATACCA GCTGGCTGCA
4201 ACTGTTTATT AAAAACACAG CACTGTGCAA ACACGAAAGT GGACGTATAC GGTGTGACGC
4261 CTGCCCGGTG CCGGAAGGTT AATTGATGGG GTTAGCGCAA GCGAAGCTCT TGATCGAAGC
4321 CCCGGTAAAC GGCGGCCGTA ACTATAACGG TCCTAAGGTA GCGAAATTCC TTGTCGGGTA
4381 AGTTCCGACC TGCACGAATG GCGTAATGAT GGCCAGGCTG TCTCCACCCG AGACTCAGTG
4441 AAATTGAACT CGCTGTGAAG ATGCAGTGTA CCCGCGGCAA GACGGAAGA CCCCGTGAAC
4501 CTTTACTATA GCTTGACACT GAACATTGAG CCTTGATGTG TAGGATAGGT GGGAGGCTTT
4561 GAAGTGTGGA CGCCAGTCTG CATGGAGCCG ACCTTGAAAT ACCACCCTTT AATGTTTGAT
4621 GTTCTAACGT TGACCCGTAA TCCGGGTTGC GGACAGTGTC TGGTGGGTAG TTTGACTGGG
4681 GCGGTCTCCT CCTAAAGAGT AACGGAGGAG CACGAAGGTT GGCTAATCCT GGTCGGACAT
4741 CAGGAGGTTA GTGCAATGGC ATAAGCCAGC TTGACTGCGA GCGTGACGGC GCGAGCAGGT
4801 GCGAAAGCAG GTCATAGTGA TCCGGTGGTT CTGAATGGAA GGGCCATCGC TCAACGGATA
4861 AAAGGTACTC CGGGGATAAC AGGCTGATAC CGCCCAAGAG TTCATATCGA CGGCGGTGTT
4921 TGGCACCTCG ATGTCGGCTC ATCACATCCT GGGGCTGAAG TAGGTCCCAA GGGTATGGCT
4981 GTTCGCCATT TAAAGTGGTA CGCGAGCTGG GTTTAGAACG TCGTGAGACA GTTCGGTCCC
5041 TATCTGCCGT GGGCGCTGGA GAACTGAGGG GGGCTGCTCC TAGTACGAGA GGACCGGAGT
5101 GGACGCATCA CTGGTGTTCG GGTTGTCATG CCAATGGCAC TGCCCGGTAG CTAAATGCGG
5161 AAGAGATAAG TGCTGAAAGC ATCTAAGCAC GAAACTTGCC CCGAGATGAG TTCTCCCTGA
5221 CCCTTTAAGG GTCCTGAAGG AACGTTGAAG ACGACGACGT TGATAGGCCG GGTGTGTAAG
5281 CGCAGCGATG CGTTGAGCTA ACCGGTACTA ATGAACCGTG AGGCTTAACC TTACAACGCC
5341 GAAGCTGTTT TGGCGGATGA GAGAAGATTT TCAGCCTGAT ACAGATTAAA TCAGAACGCA
5401 GAAGCGGTCT GATAAAACTC GAGGCATCAA ATAAAACGAA AGGCTCAGTC GAAAGACTGG
5461 GCCTTTCGTT TTATCTGTTG TTTGTCGGTG AACGCTCTCC TGAGTAGGAC AAATCCGCCG
5521 GGAGCGGATT TGAACGTTGC GAAGCAACGG CCCGGAGGGT GGCGGGCAGG ACGCCCGCCA
5581 TAAACTGCCA GGCATCAAAT TAAGCAGAAG GCCATCCTGA CGGATGGCCT TTTTGCGTTT
5641 CTACAAACTC TTCCTGTCGT CATATCTACA AGCCGGCGCG CCGGGAAATG TGCGCGGAAC
5701 CCCTATTTGT TTATTTTTCT AAATACATTC AAATATGTAT CCGCTCATGA GACAATAACC
5761 CTGATAAATG CTTCAATAAT ATTGAAAAAG GAAGAGTATG AGTATTCAAC ATTTCCGTGT
5821 CGCCCTTATT CCCTTTTTTG CGGCATTTTG CCTTCCTGTT TTTGCTCACC CAGAAACGCT
5881 GGTGAAAGTA AAAGATGCTG AAGATCAGTT GGGTGCACGA GTGGGTTACA TCGAACTGGA
5941 TCTCAACAGC GGTAAGATCC TTGAGAGTTT TCGCCCCGAA GAACGTTTTC CAATGATGAG
6001 CACTTTTAAA GTTCTGCTAT GTGGCGCGGT ATTATCCCGT GTTGACGCCG GGCAAGAGCA
6061 ACTCGGTCGC CGCATACACT ATTCTCAGAA TGACTTGGTT GAGTACTCAC CAGTCACAGA
6121 AAAGCATCTT ACGGATGGCA TGACAGTAAG AGAATTATGC AGTGCTGCAA TAACCATGAG
6181 TGATAACACT GCGGCCAACT TACTTCTGAC AACGATCGGA GGACCGAAGG AGCTAACCGC
6241 TTTTTTGCAC AACATGGGGG ATCATGTAAC TCGCCTTGAT CGTTGGGAAC CGGAGCTGAA
6301 TGAAGCCATA CCAAACGACG AGCGTGACAC CACGATGCCT GCAGCAATGG CAACAACGTT
6361 GCGCAAACTA TTAACTGGCG AACTACTTAC TCTAGCTTCC CGGCAACAAT TAATAGACTG
6421 GATGGAGGCG GATAAAGTTG CAGGACCACT TCTGCGCTCG GCCCTTCCGG CTAGCTGGTT
6481 TATTGCTGAT AAATCTGGAG CCGGTGAGCG TGGGTCTCGC GGTATCATTG CAGCACTGGG
6541 GCCAGATGGT AAGCCCTCCC GTATCGTAGT TATCTACACG ACGGGGAGTC AGGCAACTAT
6601 GGATGAACGA AATAGACAGA TCGCTGAGAT AGGTGCCTCA CTGATTAAGC ATTGGTAACT
6661 GCAGACCAAG TTTACTCATA TATACTTTAG ATTGATTTAA AACTTCATTT TTAATTTAAA

```
     6721 AGGATCTAGG TGAAGATCCT TTTTGATAAT CTCATGACCA AAATCCCTTA ACGTGAGTTT
     6781 TCGTTCCACT GAGCGTCAGA CCCCGTAGAA AAGATCAAAG GATCTTCTTG AGATCCTTTT
     6841 TTTCTGCGCG TAATCTGCTG CTTGCAAACA AAAAAACCAC CGCTACCAGC GGTGGTTTGT
     6901 TTGCCGGATC AAGAGCTACC AACTCTTTTT CCGAAGGTAA CTGGCTTCAG CAGAGCGCAG
     6961 ATACCAAATA CTGTCCTTCT AGTGTAGCCG TAGTTAGGCC ACCACTTCAA GAACTCTGTA
     7021 GCACCGCCTA CATACCTCGC TCTGCTAATC CTGTTACCAG TGGCTGCTGC CAGTGGCGAT
     7081 AAGTCGTGTC TTACCGGGTT GGACTCAAGA CGATAGTTAC CGGATAAGGC GCAGCGGTCG
     7141 GGCTGAACGG GGGGTTCGTG CACACAGCCC AGCTTGGAGC GAACGACCTA CACCGAACTG
     7201 AGATACCTAC AGCGTGAGCT ATGAGAAAGC GCCACGCTTC CCGAAGGGAG AAAGGCGGAC
     7261 AGGTATCCGG TAAGCGGCAG GGTCGGAACA GGAGAGCGCA CGAGGGAGCT TCCAGGGGGA
     7321 AACGCCTGGT ATCTTTATAG TCCTGTCGGG TTTCGCCACC TCTGACTTGA GCGTCGATTT
     7381 TTGTGATGCT CGTCAGGGGG GCGGAGCCTA TGGAAAAACG CCAGCAACGC GGCCTTTTTA
     7441 CGGTTCCTGG CCTTTTGCTG G
//
```

ptRNA100

LOCUS   Exported   4035 bp ds-DNA   circular SYN 18-JUN-2019
DEFINITION synthetic circular DNA
ACCESSION   .
VERSION   .
KEYWORDS   .
SOURCE   synthetic DNA construct
  ORGANISM synthetic DNA construct
REFERENCE   1 (bases 1 to 4035)
  AUTHORS   Alexander S. Mankin
  TITLE   Direct Submission
  JOURNAL   Exported Tuesday, Jun 18, 2019 from SnapGene 4.3.10
            https://www.snapgene.com
FEATURES   Location/Qualifiers
     source   1..4035
                     /organism="synthetic DNA construct"
                     /mol_type="other DNA"
     promoter   72..100
                     /label=tac promoter
                     /note="strong E. coli promoter; hybrid between the trp and
                     lac UV5 promoters"
     tRNA   174..250
                     /locus_tag="tRNA Asp"
                     /label=tRNA Asp
     tRNA   259..334
                     /locus_tag="tRNA Trp"
                     /label=tRNA Trp
     tRNA   373..449
                     /locus_tag="tRNA Ile"
                     /label=tRNA Ile
     tRNA   492..567
                     /locus_tag="tRNA Ala"
                     /label=tRNA Ala
     tRNA   606..681
                     /locus_tag="tRNA Glu"
                     /label=tRNA Glu
     terminator   698..740
                     /locus_tag="T1"

```
                     /label=T1
     rep_origin      1298..1843
                     /direction=RIGHT
                     /label=p15A ori
                     /note="Plasmids containing the medium-copy-number p15A
                     origin of replication can be propagated in E. coli cells
                     that contain a second plasmid with the ColE1 origin."
     CDS             3026..3817
                     /codon_start=1
                     /gene="aadA"
                     /product="aminoglycoside adenylyltransferase (Murphy,
                     1985)"
                     /label=SmR
                     /note="confers resistance to spectinomycin and
                     streptomycin"
                     /translation="MREAVIAEVSTQLSEVVGVIERHLEPTLLAVHLYGSAVDGGLKPH
                     SDIDLLVTVTVRLDETTRRALINDLLETSASPGESEILRAVEVTIVVHDDIIPWRYPAK
                     RELQFGEWQRNDILAGIFEPATIDIDLAILLTKAREHSVALVGPAAEELFDPVPEQDLF
                     EALNETLTLWNSPPDWAGDERNVVLTLSRIWYSAVTGKIAPKDVAADWAMERLPAQYQP
                     VILEARQAYLGQEEDRLASRADQLEEFVHYVKGEITKVVGK"
ORIGIN
        1 CCGCATTAAA ATCTAGCGAG GATCCGAGAT CTCTTTCCTT TCCATCAAAA AAATATTGAT
       61 GAAATGAGCT GTTGACAATT AATCATCGGC TCGTATAATG TGTGGAATTG TCACACAGGA
      121 AACAGAATTC CCGGGGATCT GGGGGATCAT CGATGGTTGT AAAAGAATTC GGTGGAGCGG
      181 TAGTTCAGTC GGTTAGAATA CCTGCCTGTC ACGCAGGGGG TCGCGGGTTC GAGTCCCGTC
      241 CGTTCCGCCA CCCTAATTAG GGGCGTAGTT CAATTGGTAG AGCACCGGTC TCCAAAACCG
      301 GGTGTTGGGA GTTCGAGTCT CTCCGCCCCT GCCAGAAATC ATCCTTGTCG ATGGGAGCAG
      361 TAAAACCTCT ACAGGCTTGT AGCTCAGGTG GTTAGAGCGC ACCCCTGATA AGGGTGAGGT
      421 CGGTGGTTCA AGTCCACTCA GGCCTACCAA ATTTGCACGG CAAATTTGAA GAGGTTTTAA
      481 CTACATGTTA TGGGGCTATA GCTCAGCTGG GAGAGCGCCT GCTTTGCACG CAGGAGGTCT
      541 GCGGTTCGAT CCCGCATAGC TCCACCATCT CTGTAGTGAT TAAGAGCGTG ATAAGCAATT
      601 TTCGTGTCCC CTTCGTCTAG AGGCCCAGGA CACCGCCCTT TCACGGCGGT AACAGGGGTT
      661 CGAATCCCCT AGGGGACGCC ACTCTAGGAA ATCCGCCATA AAACAAAAGG CTCAGTCGGA
      721 AGACTGGGCC TTTTGTTTTA TGTCGACGGG CATAAATAGG TTTAATTTTG CTACGGGGGC
      781 GTTATTTAGG TTTTTTCTTC TTTCGAAAAA ATCTTTCTTT ATGAAGTTAA AAGCTATGTA
      841 TTCAATAGCA TATTTTGAAT ATGGACATAG AATAGTGCTT ATCACTATTG CATATAGCAT
      901 CTTATCTGAC ACAAGGAAAT AATACCCTTC GCTGTTTTTT GTTATAAGGT ATATATATAT
      961 AAGTGTGCAG TACAGGCCAA ATAAAATATT TTTTATGTAG TATCTTAAAT CCCGCAAGAG
     1021 GCCCGGCAGT ACCGGCATAA CCAAGCCTAT GCCTACAGCA TCCAGGGTGA CGGTGCCGAG
     1081 GATGACGATG AGCGCATTGT TAGATTTCAT ACACGGTGCC TGACTGCGTT AGCAATTTAA
     1141 CTGTGATAAA CTACCGCATT AAAGCTTATC GATGATAAGC TGTCAAACAT GAGAATTACA
     1201 ACTTATATCG TATGGGGCTG ACTTCAGGTG CTACATTTGA AGAGATAAAT TGCACTGAAA
     1261 TCTAGAAATA TTTTATCTGA TTAATAAGAT GATCTTCTTG AGATCGTTTT GGTCTGCGCG
     1321 TAATCTCTTG CTCTGAAAAC GAAAAAACCG CCTTGCAGGG CGGTTTTTCG AAGGTTCTCT
     1381 GAGCTACCAA CTCTTTGAAC CGAGGTAACT GGCTTGGAGG AGCGCAGTCA CCAAAACTTG
     1441 TCCTTTCAGT TTAGCCTTAA CCGGCGCATG ACTTCAAGAC TAACTCCTCT AAATCAATTA
     1501 CCAGTGGCTG CTGCCAGTGG TGCTTTTGCA TGTCTTTCCG GGTTGGACTC AAGACGATAG
     1561 TTACCGGATA AGGCGCAGCG GTCGGACTGA ACGGGGGGTT CGTGCATACA GTCCAGCTTG
     1621 GAGCGAACTG CCTACCCGGA ACTGAGTGTC AGGCGTGGAA TGAGACAAAC GCGGCCATAA
     1681 CAGCGGAATG ACACCGGTAA ACCGAAAGGC AGGAACAGGA GAGCGCACGA GGGAGCCGCC
     1741 AGGGGGAAAC GCCTGGTATC TTTATAGTCC TGTCGGGTTT CGCCACCACT GATTTGAGCG
     1801 TCAGATTTCG TGATGCTTGT CAGGGGGGCG GAGCCTATGG AAAAACGGCT TTGCCGCGGC
     1861 CCTCTCACTT CCCTGTTAAG TATCTTCCTG GCATCTTCCA GGAAATCTCC GCCCCGTTCG
     1921 TAAGCCATTT CCGCTCGCCG CAGTCGAACG ACCGAGCGTA GCGAGTCAGT GAGCGAGGAA
     1981 GCGGAATATA TCCTGTATCA CATATTCTGC TGACGCACCG GTGCAGCCTT TTTTCTCCTG
     2041 CCACATGAAG CACTTCACTG ACACCCTCAT CAGTGCCAAC ATAGTAAGCC AGTATACACT
```

```
2101 CCGCTAGCGC TGATGTCCGG CGGTGCTTTT GCCGTTACGC ACCACCCCGT CAGTAGCTGA
2161 ACAGGAGGGA CAGAGCTTTA TGCTTGTAAA CCGTTTTGTG AAAAAATTTT TAAAATAAAA
2221 AAGGGGACCT CTAGGGTCCC CAATTAATTA GTAATATAAT CTATTAAAGG TCATTCAAAA
2281 GGTCATCCAC CGGATCAATT CCCCTGCTCG CGCAGGCTGG GTGCCAAGCT CTCGGGTAAC
2341 ATCAAGGCCC GATCCTTGGA GCCCTTGCCC TCCCGCACGA TGATCGTGCC GTGATCGAAA
2401 ATCCAGATCC TTGACCCGCA TTTGCAAACC CTCACTGATC CGCATGCCCG TTCCATACAG
2461 AAGCTGGGCG AACAAACGAT GCTCGCCTTC CAGAAAACCG AGGATGCGAA CCACTTCATC
2521 CGGGGTCAGC ACCACCGGCA AGCGCCGCGA CGGCCGAGGT CTTCCGATCT CCTGAAGCCA
2581 GGGCAGATCC GTGCACAGCA CCTTGCCGTA GAAGAACAGC AAGGCCGCCA ATGCCTGACG
2641 ATGCGTGGAG ACCGAAACCT TGCGCTCGTT CGCCAGCCAG GACAGAAATG CCTCGACTTC
2701 GCTGCTGCCC AAGGTTGCCG GGTGACGCAC ACCGTGGAAA CGGATGAAGG CACGAACCCA
2761 GTGGACATAA GCCTGTTCGG TTCGTAAGCT GTAATGCAAG TAGCGTATGC GCTCACGCAA
2821 CTGGTCCAGA ACCTTGACCG AACGCAGCGG TGGTAACGGC GCAGTGGCGG TTTTCATGGC
2881 TTGTTATGAC TGTTTTTTTG GGGTACAGTC TATGCCTCGG GCATCCAAGC AGCAAGCGCG
2941 TTACGCCGTG GGTCGATGTT TGATGTTATG GAGCAGCAAC GATGTTACGC AGCAGGGCAG
3001 TCGCCCTAAA ACAAAGTTAA ACATCATGAG GGAAGCGGTG ATCGCCGAAG TATCGACTCA
3061 ACTATCAGAG GTAGTTGGCG TCATCGAGCG CCATCTCGAA CCGACGTTGC TGGCCGTACA
3121 TTTGTACGGC TCCGCAGTGG ATGGCGGCCT GAAGCCACAC AGTGATATTG ATTTGCTGGT
3181 TACGGTGACC GTAAGGCTTG ATGAAACAAC GCGGCGAGCT TTGATCAACG ACCTTTTGGA
3241 AACTTCGGCT TCCCCTGGAG AGAGCGAGAT TCTCCGCGCT GTAGAAGTCA CCATTGTTGT
3301 GCACGACGAC ATCATTCCGT GGCGTTATCC AGCTAAGCGC GAACTGCAAT TTGGAGAATG
3361 GCAGCGCAAT GACATTCTTG CAGGTATCTT CGAGCCAGCC ACGATCGACA TTGATCTGGC
3421 TATCTTGCTG ACAAAAGCAA GAGAACATAG CGTTGCCTTG GTAGGTCCAG CGGCGGAGGA
3481 ACTCTTTGAT CCGGTTCCTG AACAGGATCT ATTTGAGGCG CTAAATGAAA CCTTAACGCT
3541 ATGGAACTCG CCGCCCGACT GGGCTGGCGA TGAGCGAAAT GTAGTGCTTA CGTTGTCCCG
3601 CATTTGGTAC AGCGCAGTAA CCGGCAAAAT CGCGCCGAAG GATGTCGCTG CCGACTGGGC
3661 AATGGAGCGC CTGCCGGCCC AGTATCAGCC CGTCATACTT GAAGCTAGAC AGGCTTATCT
3721 TGGACAAGAA GAAGATCGCT TGGCCTCGCG CGCAGATCAG TTGGAAGAAT TTGTCCACTA
3781 CGTGAAAGGC GAGATCACCA AGGTAGTCGG CAAATAATGT CTAACAATTC GTTCAAGCCG
3841 ACGCCGCTTC GCGGCGCGGC TTAACTCAAG CGTTAGATGC ACTAAGCACA TAATTGCTCA
3901 CAGCCAAACT ATCAGGTCAA GTCTGCTTTT ATTATTTTTA AGCGTGCATA ATAAGCCCTA
3961 CACAAATTGG GAGATATATC ATGAAAGGCT GGCTTTTTCT TGTTATCGCA ATAGTTGGCG
4021 AAGTAATCGC AACAT
//
```
