# Supplementary material for: Ribosome engineering reveals the importance of 5S rRNA autonomy for ribosome assembly
Source: Nat Commun. 2020 Jun 9;11:2900. doi: 10.1038/s41467-020-16694-8 (PMC7283268; doi:10.1038/s41467-020-16694-8)
Supplement: Supplementary file 2 — Peer Review File [file 41467_2020_16694_MOESM2_ESM.pdf]

Reviewer #1 (Remarks to the Author):

Huang et al present a comprehensive set of experiments to define the role of 5S autonomy in ribosome assembly and function. In all organisms, 5S rRNA is transcribed separately from the rest of the rRNA and is incorporated into the large ribosomal subunit during early maturation. The importance of 5S and its associated ribosomal proteins to ribosome function has been long established, but why it is maintained as an independent structural element has never been conclusively examined. Using clever molecular engineering, Huang et al were able to splice a circularly permuted 5S rRNA into the 23S rRNA at two separate positions. Using a library approach followed by subsequent selection and clone identification, they were able to isolate viable strains in which the 5S rRNA is present entirely as a non-independent component of the large ribosomal subunit. They exhaustively validate their approach using in vitro and in vivo experiments and are able to show that 5S rRNA autonomy is not required for ribosome catalytic function, but rather, allows for critical ribosome biogenesis steps essential for proper P-site formation to be carried out more effectively. This is an important finding for the field and will inform the role of 5S in large subunit maturation not only in bacterial, but in eukaryotic cells as well.

A few points:

1. In the structural figures presented in Figures 6 and 7, it is difficult to see the differences between the "normal" 70S and aberrant 50S (and 70S) subunits (i.e panels 6a and 7a). Perhaps the larger, more detailed side-by-side panels from Sup. Fig. 5 could be moved to one of the main figure panels to highlight the main difference in the ribosomes? In supplementary figure 5, it would be nice to see the map densities for the RNA elements shown in panel b so that the level of confidence in these models can be assessed by the reader (always important in the resolution range of 3 – 3.5Å.

2. In their discussion, the authors suggest that the unwinding and rearrangements of H89 in various eukaryotic intermediates containing the Nog2 GTPase hint at the importance of this process in proper PTC maturation. In this context, they should also look at the 50S intermediates with bound RbgA, the bacterial Nog2 homolog (Seffouh et al, 2019).

3. Have the authors considered purifying 23-cp5S ribosomes after a temporary temperature shift to the non-permissive temperature? These samples may reveal additional, kinetically trapped intermediate states. Similarly, have the authors tried to overexpress uL16 to see if this could increase the growth rates of DH42 cells.

Overall, this paper reveal a series of bold and clever experiments to define the role of 5S rRNA as an autonomous ribosome component. The importance of the 5S rRNP in the metazoan nuclear stress response also shows how the autonomous nature of the 5S rRNA can be exploited in other cellular pathways, thus making these findings relevant beyond bacterial ribosome biology.

Reviewer #2 (Remarks to the Author):

Almost all ribosomes in nature contain a 5S rRNA that is synthesized as a molecule separate from the other rRNAs. Thus, 5S rRNA is assembled onto large ribosomal subunits as a separate entity, and functions in ribosomes as a noncovalently associated component of the central protuberance overlooking the peptidyltransferase center. Here, Alexander Mankin and colleagues ask whether it is obligatory that 5S rRNA is "autonomous". They very cleverly engineered E. coli strains whose only form of 5S rRNA is covalently attached to 23S rRNA. These bacteria grow well at 37 degrees and exhibit seemingly fine protein synthesis. However, these strains exhibit significant defects in assembly of the peptidyltransferase center. Furthermore, these mutant strains are cold-sensitive, i.e., unable to grow at all at 30 degrees….suggesting a problem in RNA folding,

This manuscript well designed, clearly described, and for the most part comes to logical and interesting conclusions. Therefore, it is worthy of publication in Nature Communications, after some slight improvements and addressing a few questions.

(1) Abstract: The authors state that autonomous 5S rRNA is not required for cell growth. This is a little bit misleading; the mutant strain is extremely cold-sensitive (Fig.5a).
(2) Introduction: The authors state that the general role for 5S rRNA in protein synthesis is enigmatic. Yes, but either here or in the Discussion, could they discuss a little more what defects have been observed in 5S rRNA mutants? For example, see two papers from Jon Dinman's group (2005, I think) describing effects of point mutations in yeast 5S rRNA that affect fidelity of protein synthesis.
(3) Figure 1: To make this figure clearer: The highlighted dark blue r proteins are difficult to see above the background of light blue rRNA in 1b. Maybe one might color them differently from the rRNA? In addition, could the authors use a darker shade of blue for the RNA in Figure1e?
(4) Figure 2f: Could the authors comment why the ratio of mutant to wt 5SrRNA is higher in 50S subunits from DH42 than in CH84?
(5) Figure 4: Protein synthesis is assayed using as reporters two different single proteins. What is the effect on global protein synthesis? On accuracy of protein synthesis, using sensitive reporters? Can the authors make clearer by how much the efficiency of subunit assembly is decreased in the DH42 strain (Figs 5, 6 and 7)? Is the effect on assembly small enough to explain the very modest defect in protein synthesis?
(6) Page 14: Can the authors speculate, based on their cryo-EM data, why the defective 50S subunits are unable to join with 30S subunits?
(7) Figure 5-7: Here the authors show by mass spectrometry that there is a deficiency in four ribosomal proteins in the DH42* 50S subunits: uL16, bL33, bL35, and bL36. Yet, only uL16 and/or bL33 are seen to be missing from mutant particles by cryo-EM. Is this because many of the mutant particles included in the bulk MS data are damaged and not included in the sorted single molecule structural data in Fig.7?
(8) Figure 5d: Can the authors make the r proteins a different color from the 5S rRNA. All are red in this figure and are a little difficult to distinguish from the RNA.
(9) Could the authors explain in more detail where uL16, bL33, bL35, and bL36 lie in the assembly pathway?
(10) Please explain in a little more detail why there are both wt and mutant particles produced in the mutant. Are the mutant particles following a different assembly landscape pathway (the authors do suggest this I think), or is the mutant simply leaky, i.e., there is a lower probability of uL16 and bL33 entering particles in the mutant?. Because the one defect seen in this very interesting mutant is subunit assembly, I think that the manuscript would be more interesting if the authors added a little more discussion about where in the pathway assembly is failing and why, and how this compares to the findings of other groups. Can they compare their results to those for GTPase mutants described by Ortega, Williamson and Britton, and Gao, where assembly of L16, L33, L35, and L36 is also affected?
(11) Supplementary Figure 6 is unclear to me. Could the authors include separate images indicating exactly how 5S rRNA is configured in prokaryotes cf. eukaryotes?

Respectfully,
John Woolford

Reviewer #1:

Huang et al present a comprehensive set of experiments to define the role of 5S autonomy in ribosome assembly and function. In all organisms, 5S rRNA is transcribed separately from the rest of the rRNA and is incorporated into the large ribosomal subunit during early maturation. The importance of 5S and its associated ribosomal proteins to ribosome function has been long established, but why it is maintained as an independent structural element has never been conclusively examined. Using clever molecular engineering, Huang et al were able to splice a circularly permuted 5S rRNA into the 23S rRNA at two separate positions. Using a library approach followed by subsequent selection and clone identification, they were able to isolate viable strains in which the 5S rRNA is present entirely as a non-independent component of the large ribosomal subunit. They exhaustively validate their approach using in vitro and in vivo experiments and are able to show that 5S rRNA autonomy is notrequired for ribosome catalytic function, but rather, allows for critical ribosome biogenesis steps essential for proper P-site formation to be carried out more effectively. This is an important finding for the field and will inform the role of 5S in large subunit maturation not only in bacterial, but in eukaryotic cells as well.

We thank the reviewer for the kind words about our work.

A few points:

1. In the structural figures presented in Figures 6 and 7, it is difficult to see the differences between the “normal” 70S and aberrant 50S (and 70S) subunits (i.e panels 6a and 7a). Perhaps the larger, more detailed side-by-side panels from Sup. Fig. 5 could be moved to one of the main figure panels to highlight the main difference in the ribosomes? In supplementary figure 5, it would be nice to see the map densities for the RNA elements shown in panel b so that the level of confidence in these models can be assessed by the reader (always important in the resolution range of 3 – 3.5Å.

This is a valid point, which unfortunately is difficult to address because the overall structure of the ‘good’ mutant and wt ribosomes are essentially the same. Nevertheless, to better illustrate the difference between the wt and mutant ribosomes, we have modified the main Figure 6 by adding to it a new panel that depicts the local difference in the structures at the site of attachment of the 5S rRNA to the 23S rRNA. In the originally submitted paper this was a part of Supplementary Figure 4. In addition, to further improve the comparison, we show the wt and mutant structures side-by-side rather than overlaying them (as in the original supplementary figure 4b).
The purpose of Figure 7 is to demonstrate that even though the main architecture of the aberrant 50S subunit remains the same, the RNA in the PTC is misfolded. To better highlight this point, we now show side-by-side the similarity of the overall structures of the wt and mutant aberrant 50S subunits (panel a) and the dramatic difference in the rRNA structure in the PTC (panel c). We believe adding more panels to this figure would overburden it.
Following the reviewer’s request, we have added a new panel to Supplementary Figure 5 (panel b) that shows cryo-EM densities for the misfolded rRNA in the aberrant 50S subunits.

2. In their discussion, the authors suggest that the unwinding and rearrangements of H89 in various eukaryotic intermediates containing the Nog2 GTPase hint at the importance of this

process in proper PTC maturation. In this context, they should also look at the 50S intermediates with bound RbgA, the bacterial Nog2 homolog (Seffouh et al, 2019).

We thank the reviewer for bringing RbgA to our attention. We also want to apologize for inadvertently misleading the reviewer due to a typo, as it is not Nog2 but Nog1 that is directly involved in the strand separation of H89 during maturation of the eukaryotic large ribosomal subunit. This said, Nog2 and Nog1 directly interact with each other suggesting that Nog2 and its bacterial homolog RbgA can play an indirect role in the positioning of H89. In the revised manuscript we added two new RbgA-related references and we now write:
"Curiously, the unwinding of H89 that we observed in the aberrant 50S subunits is reminiscent of the *Nog1*-mediated separation of H89 strands in the assembly intermediates of the eukaryotic large ribosomal subunit where eventual rearrangement of H89 into its mature conformation is required for uL16 binding {Wu, 2016 #9095;Kargas, 2019 #9123}. *In eukaryotes, Nog1 interacts with another assembly factor, Nog2 {Wu, 2016 #9095}, which is homologous to the bacterial protein RbgA. Recent cryo-EM reconstructions of the Bacillus subtilis large ribosomal subunit assembly intermediates in the rgbA knock-out strain reveal local disruption of the PTC, including H89 {Seffouh, 2019 #9216}. Interestingly, it has been proposed that the release of RbgA and the subsequent maturation of the 50S requires the presence of uL16 {Jomaa, 2014 #8764}."*

3. Have the authors considered purifying 23-cp5S ribosomes after a temporary temperature shift to the non-permissive temperature? These samples may reveal additional, kinetically trapped intermediate states. Similarly, have the authors tried to overexpress uL16 to see if this could increase the growth rates of DH42 cells.

Isolating assembly intermediates after temperature downshift is an interesting idea. We have not considered carrying out these experiments, partly because the analysis of multiple assembly intermediates would require a much deeper structural analysis than the one carried out at this stage of the project. Nevertheless, this could be a possible future direction. As to overexpressing uL16, it is also an interesting thought. However, it is equally possible that overexpression of not the poorly incorporated r-proteins, but of some assembly factors could be even more beneficial. Selecting faster growing mutants, either by introducing the ASKA library of plasmids encoding each of the *E. coli* proteins or selecting spontaneous chromosomal mutants is on our 'to do' list for future studies.

Overall, this paper reveal a series of bold and clever experiments to define the role of 5S rRNA as an autonomous ribosome component. The importance of the 5S rRNP in the metazoan nuclear stress response also shows how the autonomous nature of the 5S rRNA can be exploited in other cellular pathways, thus making these findings relevant beyond bacterial ribosome biology.

Once again, we thank the reviewer for the high assessment of our work and thoughtful suggestions.

Reviewer #2 (Remarks to the Author):

Almost all ribosomes in nature contain a 5S rRNA that is synthesized as a molecule separate from the other rRNAs. Thus, 5S rRNA is assembled onto large ribosomal subunits as a separate entity, and functions in ribosomes as a noncovalently associated component of the central

protuberance overlooking the peptidyltransferase center. Here, Alexander Mankin and colleagues ask whether it is obligatory that 5S rRNA is "autonomous". They very cleverly engineered E. coli strains whose only form of 5S rRNA is covalently attached to 23S rRNA. These bacteria grow well at 37 degrees and exhibit seemingly fine protein synthesis. However, these strains exhibit significant defects in assembly of the peptidyltransferase center. Furthermore, these mutant strains are cold-sensitive, i.e., unable to grow at all at 30 degrees….suggesting a problem in RNA folding,

This manuscript well designed, clearly described, and for the most part comes to logical and interesting conclusions. Therefore, it is worthy of publication in Nature Communications, after some slight improvements and addressing a few questions.

We thank the reviewer for reading carefully our manuscript and for enthusiastic feedback regarding the quality and importance of our work.

(1) Abstract: The authors state that autonomous 5S rRNA is not required for cell growth. This is a little bit misleading; the mutant strain is extremely cold-sensitive (Fig.5a).

In the revised manuscript we clarify that autonomous 5S rRNA is not required for *E. coli* growth under standard growth conditions.

(2) Introduction: The authors state that the general role for 5S rRNA in protein synthesis is enigmatic. Yes, but either here or in the Discussion, could they discuss a little more what defects have been observed in 5S rRNA mutants? For example, see two papers from Jon Dinman's group (2005, I think) describing effects of point mutations in yeast 5S rRNA that affect fidelity of protein synthesis.

We have extended the discussion of possible functions of 5S rRNA (p. 3) and added several references, including the two papers from the Dinman lab suggested by the reviewer.

(3) Figure 1: To make this figure clearer: The highlighted dark blue r proteins are difficult to see above the background of light blue rRNA in 1b. Maybe one might color them differently from the rRNA? In addition, could the authors use a darker shade of blue for the RNA in Figure1e?

We have altered the presentation of the large ribosomal subunit in Fig. 1b in order to better distinguish r-proteins from rRNA.

(4) Figure 2f: Could the authors comment why the ratio of mutant to wt 5SrRNA is higher in 50S subunits from DH42 than in CH84?

The reviewer is correct that in this particular DH42 clone, the mutant 23S-5S rRNA content is higher in 50S subunits than in 70S ribosomes or polysomes, potentially implying particularly inefficient assembly. However, this is not surprising because in this experiment we analyzed just one randomly picked clone from a library of clones varying in the linker composition, a clone which apparently happened to have a suboptimal combination of linkers. In the subsequent experiments, better combinations of linkers were selected (see Fig. 3d). The most important information that we gained from the experiments with the cells with the mixed population of wt and mutant ribosomes is that ribosomes with the 23S-5S hybrid rRNA are capable to participate in translation, as inferred from their presence in the polysome fraction.

(5) Figure 4: Protein synthesis is assayed using as reporters two different single proteins. What

is the effect on global protein synthesis? On accuracy of protein synthesis, using sensitive reporters? Can the authors make clearer by how much the efficiency of subunit assembly is decreased in the DH42 strain (Figs 5, 6 and 7)? Is the effect on assembly small enough to explain the very modest defect in protein synthesis?

These are important questions, but it is hard to accurately address them experimentally. In the cell-free experiments we used purified tight-coupled ribosomes because we wanted to uncouple the translation capacity of the fully assembled ribosomes from assembly defects. By contrast, interpreting the results of measuring cellular translation efficiency or accuracy would be challenging due to the presence of functional ribosomes and misassembled ribosomes, possibly stalled at different assembly steps. For that same reason, quantifying the assembly defects is difficult and beyond the scope of this study. Analyzing the accuracy of protein synthesis catalyzed by the 23S-cp5S ribosomes, as well as its other properties (e.g. the translocation rate, the rate of peptide bond formation, etc.,) could be explored in future studies.

(6) Page 14: Can the authors speculate, based on their cryo-EM data, why the defective 50S subunits are unable to join with 30S subunits?

We report that defective 50S subunits are apparently able to join with 30S subunits forming a vacant 70S ribosomes(cryo-EM structure in Fig. S5a). However, these ribosomes do not contain tRNA and are likely less stable that the ribosomes that contain a fully assembled 50S subunit and tRNA. In the manuscript (p.14), we offer the explanation for this observation: because the interaction of the P-site tRNA with the misassembled 50S subunit is dramatically disrupted, the tRNA is unable to stabilize the defective 70S ribosome.

(7) Figure 5-7: Here the authors show by mass spectrometry that there is a deficiency in four ribosomal proteins in the DH42* 50S subunits: uL16, bL33, bL35, and bL36. Yet, only uL16 and/or bL33 are seen to be missing from mutant particles by cryo-EM. Is this because many of the mutant particles included in the bulk MS data are damaged and not included in the sorted single molecule structural data in Fig.7?

The reviewer is absolutely right: during preparation of the sample for the cryo-EM analysis we noted some aggregation. The aggregates, which likely contained the most malformed particles lacking additional ribosomal proteins, were discarded by centrifugation. Furthermore, even those aggregated (and thus badly malformed) 50S subunits that made it to the cryo-EM grid would be ignored during particle picking for image analysis. We clarified this point in the revised manuscript (p.13).

(8) Figure 5d: Can the authors make the r proteins a different color from the 5S rRNA. All are red in this figure and are a little difficult to distinguish from the RNA.

We needed to keep the color of the proteins and of 5S rRNA unchanged for the consistency with the other figures (e.g. Fig. 7d) and with the chosen palette. However, in order to unclutter the image and thus, to make proteins and 5S rRNA better distinguishable, we have converted the background 23S rRNA to a ‘spaghetti’ presentation, which we believe helps to distinguish the proteins from rRNA.

(9) Could the authors explain in more detail where uL16, bL33, bL35, and bL36 lie in the assembly pathway?

All these proteins associate with the large subunit at the late assembly steps. It is indicated on p.10 of the manuscript: “By contrast, the unassociated 50S subunits lacked not only bL33 but also a subset of other late-assembly proteins {Röhl, 1982 #1684}, most notably the essential protein uL16 {Shoji, 2011 #7742}, as well as bL35 and bL36 (Fig. 5c and Supplementary Fig. 2).”

(10) Please explain in a little more detail why there are both wt and mutant particles produced in the mutant. Are the mutant particles following a different assembly landscape pathway (the authors do suggest this I think), or is the mutant simply leaky, i.e., there is a lower probability of uL16 and bL33 entering particles in the mutant?. Because the one defect seen in this very interesting mutant is subunit assembly, I think that the manuscript would be more interesting if the authors added a little more discussion about where in the pathway assembly is failing and why, and how this compares to the findings of other groups. Can they compare their results to those for GTPase mutants described by Ortega, Williamson and Britton, and Gao, where assembly of L16, L33, L35, and L36 is also affected?

In fact, there are no wt particles in the mutant: the engineered strain that was used for most of the experiments described in our paper expresses ONLY mutant (23S-cp5S) ribosomes. As to the assembly of the latter, the available experimental results do not allow distinguishing the stalled on-pathway assembly intermediates from the dead-end particles. In particular, because the assembly intermediate that we observe with the mutant 23S-cp5S subunit is apparently unique and does not match any of the intermediates detected for the wt 50S subunit. We have added a brief discussion of this point on p.14 of the manuscript.

(11) Supplementary Figure 6 is unclear to me. Could the authors include separate images indicating exactly how 5S rRNA is configured in prokaryotes cf. eukaryotes?

The detailed structural data for the 5S complex rotation is available only for the eukaryotic 60S subunit. However, in order to clarify the message of this figure, we replaced modeled bacterial intermediate with the ‘real’ yeast structure and added one more image, that of the bacterial wt 50S subunit. It shows that in the mature 60S and 50S subunits, the position of 5S rRNA is very similar.

Respectfully,
John Woolford

We want to once again thank Dr. Woolford for his advice which helped to improve our manuscript.
